# Cocrystals of 1,2-Diiodotetrafluorobenzene with Pyridine Derivatives: Pyridine Nitrogen as a Bifurcated Acceptor with Ortho-Diiodo Halogen Bond Donors

**DOI:** 10.1021/acs.cgd.5c01439

**Published:** 2025-12-23

**Authors:** Nikola Bedeković, Antonio Magnabosco, Vladimir Stilinović, Dominik Cinčić

**Affiliations:** Department of Chemistry, Faculty of Science, 117036 University of Zagreb, Horvatovac 102a, 10000 Zagreb, Croatia

## Abstract

The behavior of 1,2-diiodotetrafluorobenzene (**12tfib**) as a halogen bond donor was studied by cocrystallizing it with a series of aromatic nitrogen bases covering a wide range of p*K*_a_ values (2.10 ≤ p*K*_a_ ≤ 9.60) and comparing the results with those reported for other perfluorinated iodobenzenes. The cocrystal screening was performed by grinding **12tfib** and each of the selected bases in a 1:2 donor:acceptor stoichiometric ratio as well as crystallization from solution in a small excess of the acceptor (ratio 1:2.5). Of the 14 bases used in this study, three weakest bases (p*K*_a_ below ca. 4) have failed to produce cocrystals, nine intermediate bases (4.85 ≤ p*K*_a_ ≤ 6.72) have yielded four new phases, which were only obtainable in grinding experiments and not as pure samples, four 1:1 cocrystals and one 1:2 cocrystal, while the two strongest bases (p*K*_a_ above ca. 7.5) have yielded 1:2 cocrystals. A total of eight new solids were studied by single-crystal X-ray diffraction. The three 1:2 cocrystals comprise discrete halogen-bonded trimers with **12tfib** acting as a ditopic halogen bond donor to two base molecules. Among the five 1:1 cocrystals, in two, **12tfib** was found to act as a monotopic donor; however, in other three, **12tfib** was found to be ditopic, with the nitrogen atom of the base being a bifurcated acceptor of a pair of halogen bonds. This highly unusual binding motif has been further investigated by quantum chemical calculations and a detailed CSD survey. The computational study has found that a binding site comprising two converging iodine atoms possesses a wide, single minimum potential, which allows for a more favorable binding of weakly and intermediately basic pyridine (aromatic sp^2^) nitrogen atoms than a single iodine atom. The CSD survey has shown that the aromatic sp^2^ nitrogen atom acting as a bifurcated halogen acceptor is indeed an extremely rare occurrence (appearing in only ca. 2.2% of the total) but considerably more likely to occur in the presence of *ortho*-diiodo halogen bond donors.

## Graphical abstract

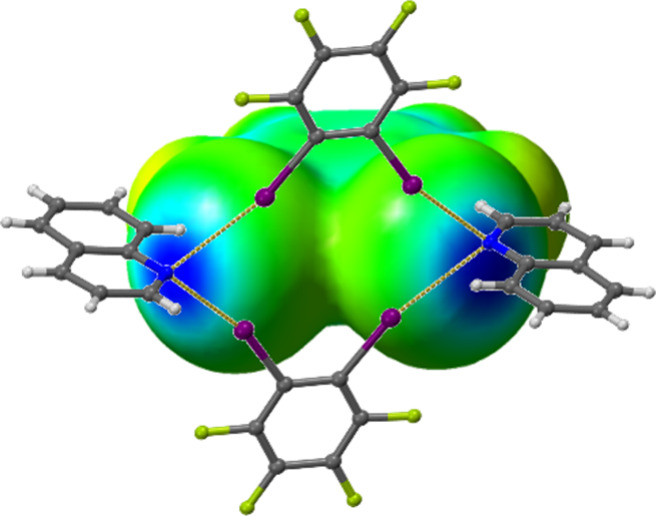

## Introduction

Despite the fact that the history of halogen bonds spans over two centuries,^1,2^ it has not become one of the central topics of study in supramolecular chemistry until the 1990s.^3–6^ Particularly important for the study of halogen bonding in the solid state was the introduction of perfluorinated iodoalkanes and arenes as halogen bond donor building blocks by Resnati and Metrangolo.^7–10^ Even though numerous other families of XB donors have been developed and studied over the years (such as iodoalkines,^11–16^ *N*-haloimides,^17–21^ halogenated cations,^22–31^ etc.), perfluorinated iodoarenes remain among the most commonly used (and commercially available) halogen bond donors in crystal engineering to date in both organic^3^ and metal–organic supramolecular chemistry.^32^ The most commonly used halogen bond donor from this group is 1,4-tetrafluorodiiodobenzene (**14tfib**), first employed for design of cocrystals a quarter of a century ago by the Dehnicke group,^33^ which accounts for 857 entries in the CSD^34^ to date (corresponding to ca. 52% of all cocrystals involving perfluorinated iodobenzenes). More recently, two other perfluoro iodobenzene analogues that are drawing considerable attention are 1,3,5-triiodo-2,4,6-trifluorobenzene (**135titfb**), a potential tritopic donor accounting for 384 entries in the CSD since its first appearance in 2007,^35^ and 1,3-tetrafluorodiiodobenzene (**13tfib**), a nonlinear ditopic donor (also first reported in 2007,^36^ but with only 105 entries).^37–39^ The fourth commonly employed halogen bond donor that belongs to this family is 1,2-tetrafluorodiiodobenzene (**12tfib**). However, in spite of its early appearance in 2001 ^40^ almost as early as **14tfib**, its occurrence in halogen-bonded cocrystals is much scarcer with only 136 CSD entries to date (accounting for mere 8.6% of cocrystals involving perfluorinated iodoarene halogen bond donors).

The relatively low occurrence of cocrystals of **12tfib** might be attributed to two possible reasons: it might be less prone to form cocrystals than **14tfib**, resulting in fewer successful cocrystallization attempts, or it has drawn less attention as a halogen bond donor because it is difficult to predict the outcome of its use as a halogen bond donor. Both issues have been observed in the case of **13tfib**, which will not form cocrystals with all acceptors with which **14tfib** will, despite its similar MEP values on σ-holes on iodine atoms.^37^ Also, it will act much more rarely as a ditopic acceptor than its **14tfib** counterpart (except in the case of cocrystals with ditopic acceptors in which it forms infinite supramolecular chains),^41^ so that the stoichiometry of the product is much more difficult to predict. Both these observations have been attributed to packing effects (easier packing of linear (**acceptor**)_2_(**14tfib**) supramolecular complexes than the bent (**acceptor**)_2_(**13tfib**) complexes).^37^ Similarly, **135titfb** very rarely acts as a tritopic donor, in spite of having the available iodine donor atoms able to participate in multiple halogen bonding, which can be attributed to anticooperativity of multiple halogen bonds formed by the same donor molecule.^35,39,42^ Both packing and anticooperativity effects can be expected to be present in cocrystals of **12tfib**, possibly even to a greater extent. Additionally, the close angular proximity of the two donor sites (60°) makes it more probable that binding of two acceptor molecules could be sterically hindered, which can affect the possibility of the formation of the desired cocrystal, leading to deviations from the expected stoichiometries and geometries of the formed supramolecular complexes.

We have decided therefore to conduct an extensive and systematic screening of **12tfib** as a potential halogen bond donor for preparation of cocrystals with monotopic nitrogen heterocycles in a wide range of basicities (Scheme 1). These acceptor molecules were chosen since focusing on monotopic heterocyclic acceptors should allow us to isolate the supramolecular behavior of **12tfib** for other possible effects. Also, as cocrystals of the majority of these bases with other perfluorinated benzenes (**13tfib**, **14tfib**, and **135tfib**) have been synthesized (or at least syntheses were attempted) by our group, this should enable us to quantitatively compare the cocrystal formation ability of **12tfib** with that of its analogues (Table 1). This study has also led us to a (rather unexpected) observation of the proclivity of *ortho*-diiodo halogen bond donors to induce the acceptor (nitrogen) atom to act as a bifurcated halogen bond acceptor, which was further investigated by using CSD data mining and quantum chemical computations.

**Scheme 1:**
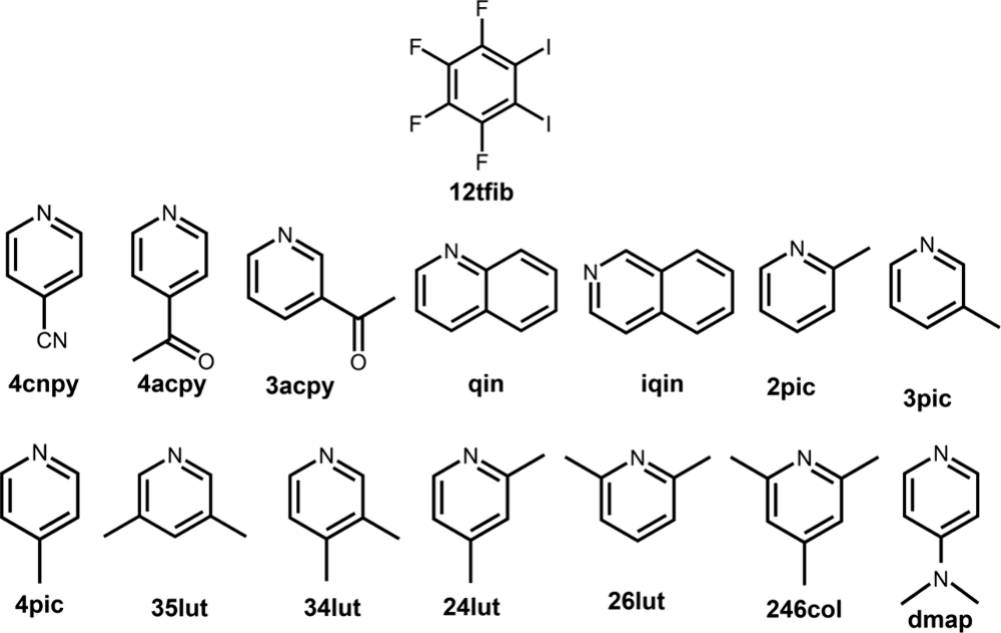
Halogen Bond Donors and Acceptors Used in the Study

**Table 1:** Donor and Acceptor (D:A) Ratios in the Cocrystals Obtained with **12tfib** and the Selected Pyridine Bases, and Comparison with Cocrystals of **13tfib**,^35^ **14tfib**,^35^ and **135tfib** ^40^

base	p*K*_a_	**12tfib**	**13tfib**	**14tfib**	**135tfib**
**4cnpy**	2.10		1:1 (NUBTAI)	1:1 (NUBSEL)	1:1 (ZEBNED)
**4acpy**	3.50			1:2 (ZEBLEB)	1:2 (ZEBNAZ)
**3acpy**	3.82			1:2 (ZEBLAX)	1:1 (ZEBMOM)
**qin**	4.85	**1:1**	1:2 (WIGTEO)	1:2 (WIGSAJ)	1:1 (ZEBPEF)
**iqin**	5.41		1:1 (WIGSEN)	1:2 (WIGRIQ)	1:2 (ZEBPAP)
**2pic**	5.97			1:2 (WIGTOY)	1:3 (ZEBMEC)
**3pic**	5.68	**1:1**		1:2 (WIGVEQ)	1:3 (ZEBMUS)
**4pic**	6.02			1:2 (WIGTUE)	
**35lut**	6.24	**1:1**	1:1 (WIHDOJ)	1:2 (WIGRAI)	1:3 (ZEBMIG)
**34lut**	6.28	**1:2**	1:1 (ZEBKUQ)	1:2 (ZEBLOL)	1:3 (ZEBNIH)
**24lut**	6.46		1:1 (ZEBKIE)	1:2 (ZEBLIF)	1:2 (ZEBMAY)
**26lut**	6.72	**1:1**	1:1 (ZEBKOK)		
**246col**	7.48	**1:2**	1:1 (WIGREM)	1:1 (WIGROW)	1:3 (ZEBLUR)
**dmap**	9.60	**1:1** (OJIMAX)	1:2 (RUYHOJ)	1:2 (RUYHID)	1:3 (RUYJAX)
		**1:2^a^**			
		**2:3^a^**			

a Two 1:2 polymorphs but with different halogen-bonded species (α – 1:2 and β – 2:3) prepared in this study.

## Results and Discussion

The tendency of **12tfib** to form cocrystals with selected halogen bond acceptors was investigated by attempted mechanochemical screening in a 1:2 donor:acceptor stoichiometric ratio (so as to enable the formation of cocrystals with a stoichiometry of 1:1 or 1:2) with the selected 14 aromatic nitrogen bases (Scheme 1). In seven cases (**3pic**, **quin**, **26lut**, **246col**, **34lut**, **35lut**, **dmap**), the grinding experiments (Table 1) resulted in novel crystalline phases, in additional three (**2pic**, **4pic**, **iqin**), a novel phase was formed, but it was mixed with an excess of **12tfib**; while with **26lut**, the product was an amorphous solid with no traces of either reactant. Grinding of **4cnpy** with **12tfib** did not result in the formation of new phases, and only mixtures of the starting reactants were isolated. Grinding of **3acpy** and **4acpy** with **12tfib** resulted in the formation of a liquid phase, which did not solidify even after several days of cooling at −15 °C. Cocrystallization of the selected acceptors with **12tfib** was also carried out by crystallization from solution, resulting in the formation of single crystals of seven new solids, whose molecular and crystal structures were determined by single-crystal X-ray diffraction (SCXRD): (**12tfib**)­(**3pic**), (**12tfib**)­(**quin**), (**12tfib**)­(**26lut**), (**12tfib**)­(**34lut**)_2_, (**12tfib**)­(**35lut**), (**12tfib**)­(**246col**)_2_, and (**12tfib**)­(**dmap**)_2_.

Cocrystals that were prepared as single crystals in the course of the present study were crystallized in two different donor:acceptor ratios—1:1 (in 4 cases) and 1:2 (in 3 cases). The 1:2 cocrystals were obtained only with bases with p*K*_a_ > 6.2, while with the weakest bases (p*K*_a_ < 4), no cocrystal formation was detected. It should be noted that the strongest base used in our study (**dmap**, with p*K*_a_ = 9.60) was reported to form a 1:1 cocrystal with **12tfib**,^43^ but this cocrystal was obtained by mixing the reactants in a 1:1 donor:acceptor stoichiometric ratio. In this study, when they were mixed in a 1:2 ratio, the 1:2 cocrystal was observed to form. The intermediate bases formed either 1:1 cocrystals or apparently unstable cocrystals (of undetermined donor:acceptor ratios), as described above.

When compared to other perfluorinated iodobenzenes, it can be seen that the general trend for stronger bases to form cocrystals with higher pyridine:donor ratios is similarly pronounced in **135tfib** cocrystals, although not as obviously among the cocrystals derived from **14tfib** and **13tfib**. The appearance of a 1:1 cocrystal with **dmap** appears to be unique to **12tfib**; for all other donors in the series, only the cocrystals with the maximal content of **dmap** (1:2 with **14tfib** and **13tfib**; 1:3 with **135tfib**) were formed. It is also interesting to note that in spite of potential steric hindrance in **12tfib**, there does not seem to be a pronounced difficulty in forming cocrystals with pyridine bases, as the number of new phases (11) obtained in the cocrystal screening is only slightly lower than the number of cocrystals successfully obtained from **14tfib** (13) and **135tfib** (12) and higher than that obtained from **13tfib** (9), using the same set of 14 pyridine bases.

In the three 1:2 cocrystals (with **246col**, **34lut**, and **dmap** as acceptors), both donor atoms of the **12tfib** molecule were employed in halogen bonding (Figure 1a–c). Due to the spatial proximity of the two donor atoms in **12tfib** and the presence of the methyl groups in **246col**, **34lut**, and **dmap**, the formed supramolecular trimers in the corresponding cocrystals are not planar (Figure 1d–f). The lutidine molecules in the cocrystal of (**12tfib**)­(**34lut**)_2_ are both rotated by approximately 13° around the halogen bond axis, which significantly reduces the repulsive interactions between the two bound pyridines and increases the stability of the resulting supramolecular complex. A somewhat more drastic deviation from the planarity of the supramolecular complexes was found in the cocrystals of (**12tfib**)­(**246col**)_2_ and α-(**12tfib**)­(**dmap**)_2_. In those cocrystals, one of the bonded acceptor molecules is rotated by 40° and 33° out of the plane of the **12tfib** molecule, respectively (Figure 1e,f), which adversely affects corresponding halogen bond angles (φ­(C–I···N)_(**12tfib**)(**246col**)2_ = 172.4° and φ­(C–I···N)_(**12tfib**)(**dmap**)2_ = 164.2°). Due to the somewhat larger available space near the second acceptor atom, the other acceptor molecules are almost coplanar with the donor molecule, and the formed halogen bond is more linear (φ­(C–I···N)_(**12tfib**)(**246col**)2_ = 177.5° and φ­(C–I···N)_(**12tfib**)(**dmap**)2_ = 175.6°). The greater mutual distance of the two acceptor molecules in (**12tfib**)­(**246col**)_2_ results in a smaller difference in the corresponding halogen bond lengths (Δ*d*_XB_ = 0.007 Å), than is the case in (**12tfib**)­(**34lut**)_2_ where the acceptor molecules are closer to each other (Δ*d*_XB_ = 0.056 Å). In α-(**12tfib**)­(**dmap**)_2_, this difference is somewhat greater than in (**12tfib**)­(**246col**)_2_ (Δ*d*_XB_ = 0.030 Å), which can be attributed to the strong anticooperativity effect, which is expected to be most pronounced in cocrystals with **dmap** as the strongest base in the range of the used acceptors.^39^ In the second formula unit of α-(**12tfib**)­(**dmap**)_2_, both acceptor molecules are rotated by 17.6° out of the mean plane of the donor molecule and achieve different halogen bond lengths (Δ*d*_XB_ = 0.248 Å). This relatively large difference can be attributed to the unfavorable sterical hindrances between two bonded acceptor molecules as well as strong anticooperativity effect.

**Figure 1:**
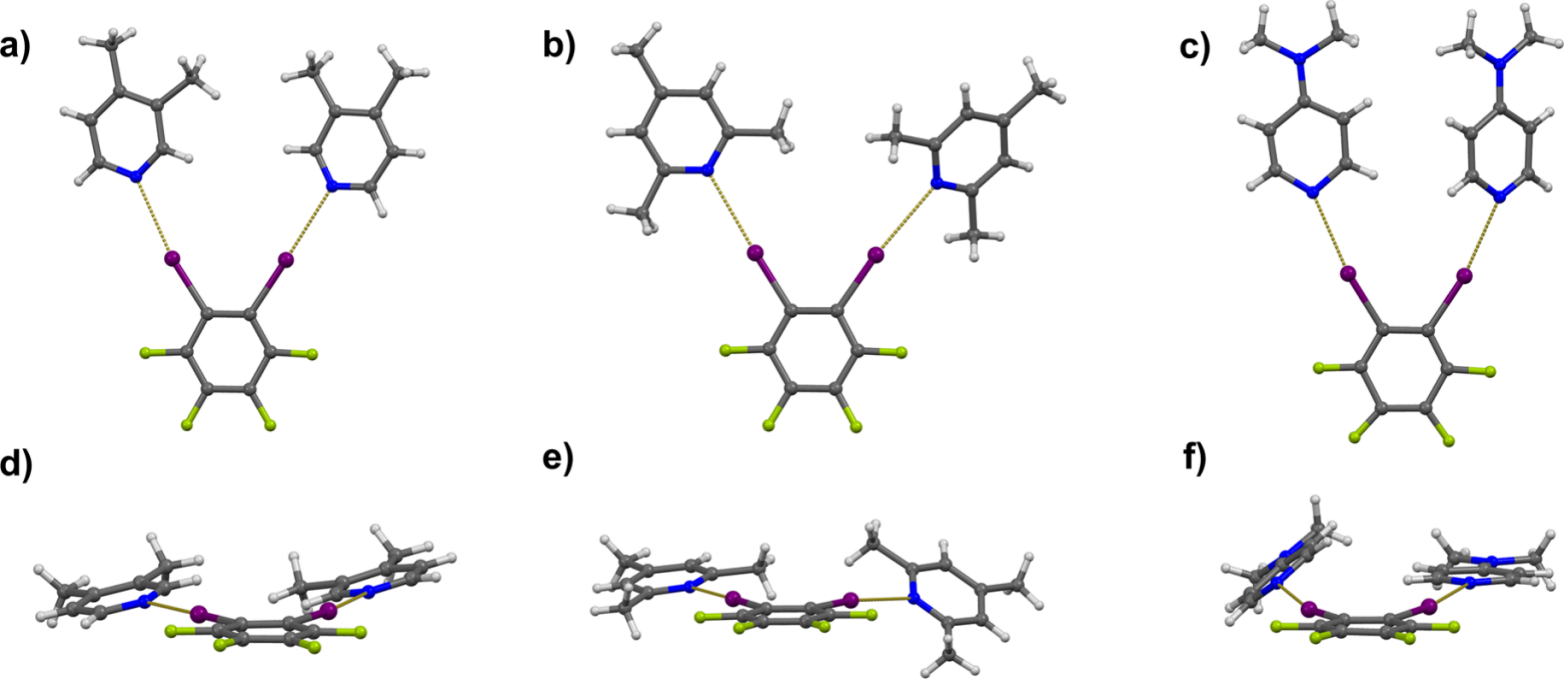
Halogen-bonded supramolecular complexes of (a), (d) (**12tfib**)­(**34lut**)_2_, (b), (e) (**12tfib**)­(**24col**)_2_, and (c), (f) (**12tfib**)­(**dmap**)_2_, shown in different orientations.

Despite the aforementioned conformational differences of the supramolecular complexes in (**12tfib**)­(**34lut**)_2_, (**12tfib**)­(**246col**)_2_, and α-(**12tfib**)­(**dmap**)_2_, which minimize mutual unfavorable steric hindrance between bonded acceptors, there is still significant contribution of the repulsive interactions between them. This is evident from the calculated values of the repulsive interaction energy term (*E*_rep_) between two acceptor molecules, which are halogen-bonded to the same donor on geometries as found in corresponding crystal structures: *E*_rep_(**34lut**···**34lut**) = +6.1 kJ mol^–1^; *E*_rep_(**246kol**···**246kol**) = +4.8 kJ mol^–1^; and *E*_rep_(**dmap**···**dmap**) = +5.2 kJ mol^–1^.

Strong bases **3pic**, **26lut**, and **35lut**, as well as a moderately strong base **quin**, form cocrystals with **12tfib** that are of 1:1 stoichiometry. In the crystal structures of those compounds, two different halogen-bonded supramolecular motives and topicities of the donor molecule were observed. In cocrystals (**12tfib**)­(**35lut**) and (**12tfib**)­(**26lut**), one acceptor molecule is halogen-bonded to one of the iodine donor atoms of **12tfib**, while the second iodine atom participates in the linking of two supramolecular complexes by I···F contacts (Figure 2). Consequently, in both cocrystals, **12tfib** is a monotopic halogen bond donor. Despite similar halogen-bonding patterns in crystal structures, the halogen bond in (**12tfib**)­(**26lut**) is somewhat longer (*d*(I···N) = 2.913 Å) than that in (**12tfib**)­(**35lut**) (*d*_1_(I···N) = 2.819 Å; *d*_2_(I···N) = 2.842 Å). This observation can be attributed to the relatively higher sterical interference of the two *ortho* methyl groups of **26lut** with the **12tfib** molecule in the donor···acceptor complex, than is the case in (**12tfib**)­(**35lut**).

**Figure 2:**
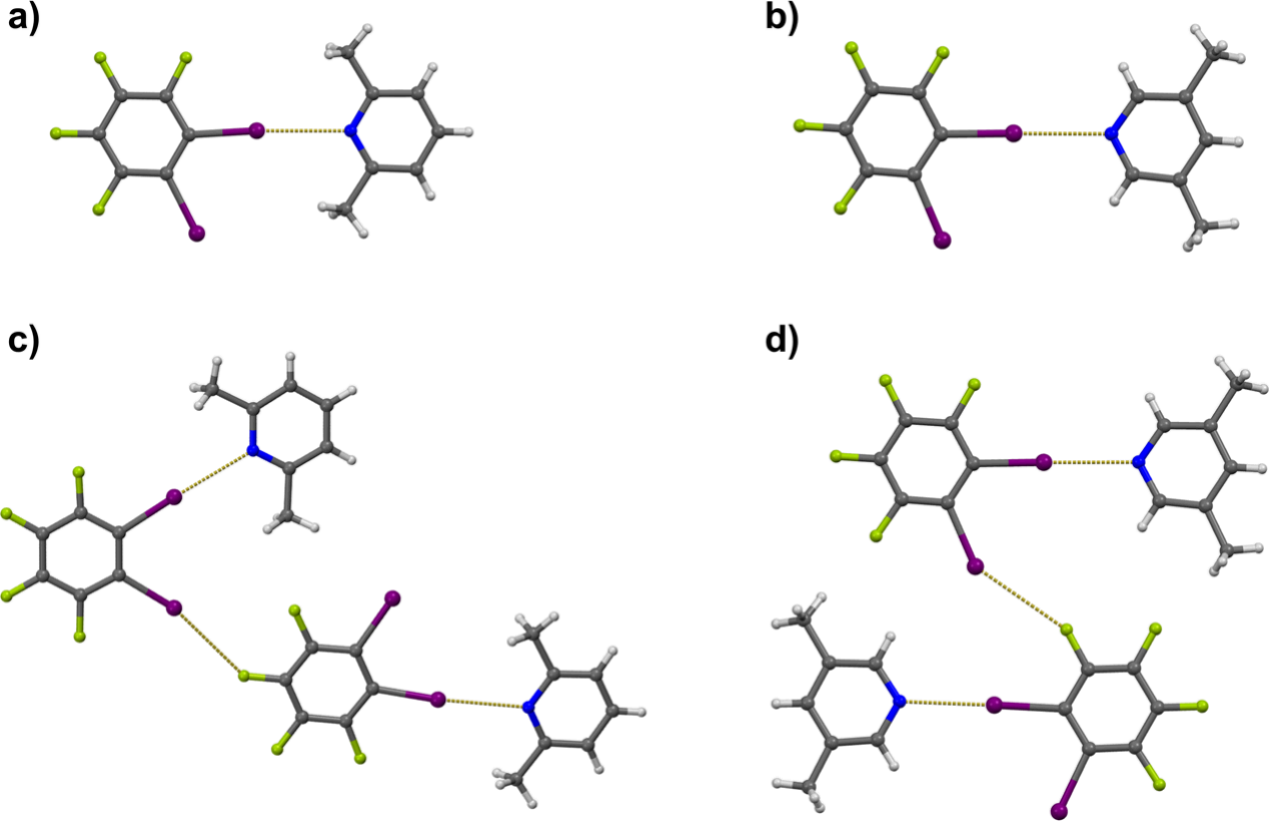
Supramolecular halogen-bonded complexes in crystal structures of (a) (**12tfib**)­(**26lut**) and (b) (**12tfib**)­(**35lut**) and their interconnection by interhalogen contacts in (c) (**12tfib**)­(**26lut**) and (d) (**12tfib**)­(**35lut**).

A somewhat different supramolecular motive has been noticed in (**12tfib**)­(**3pic**), (**12tfib**)­(**quin**), and (**12tfib**)_2_(**dmap**)_3_ cocrystals in which a bifurcated N­(sp^2^) atom was observed as the halogen bond acceptor (this is quite surprising because bifurcated halogen bonds mainly occur in cocrystals where the acceptors are oxygen^36,44^ or sulfur atoms^45^). In the crystal structure of (**12tfib**)­(**3pic**), there are two symmetrically nonequivalent **3pic** and **12tfib** molecules, of which one **3pic** molecule serves as a bifurcated halogen bond acceptor from two **12tfib** molecules, while the other (terminal) **3pic** is halogen-bonded to one of the remaining iodine atoms (Figure 3a). In such a supramolecular arrangement, one of the **12tfib** molecules is a mono- and the other is a ditopic halogen bond donor. Bifurcated I···N···I halogen bonds are asymmetric (*d*_1_ = 3.059 Å; φ_1_(C–I···N) = 174.6° and *d*_2_ = 3.047 Å; φ_2_(C–I···N) = 167.2°) and both of them are quite longer (and less linear) than the I···N halogen bond toward the terminal **3pic** molecule (*d*_1_ = 2.775 Å; φ­(C–I···N) = 176.6°). Two such supramolecular complexes are interconnected into a centrosymmetric octamer by two type II iodine···iodine interhalogen contacts (Figure 3b). In the crystal structure of (**12tfib**)­(**quin**), donor and acceptor molecules are assembled into a centrosymmetric tetramer by four I···N halogen bonds (Figure 3c). In the formed tetramer, the **12tfib** is a ditopic donor, while the **quin** molecule is a bifurcated acceptor. Like in (**12tfib**)­(**3pic**), bifurcated I···N···I halogen bonds are asymmetric (*d*_1_ = 2.977 Å; φ_1_(C–I···N) = 166.6° and *d*_2_ = 3.136 Å; φ_2_(C–I···N) = 165.8°), with the angle between the two halogen bonds slightly larger (φ­(I···N···I) = 84.5°) than that in (**12tfib**)­(**3pic**) (φ­(I···N···I) = 82.5°).

**Figure 3:**
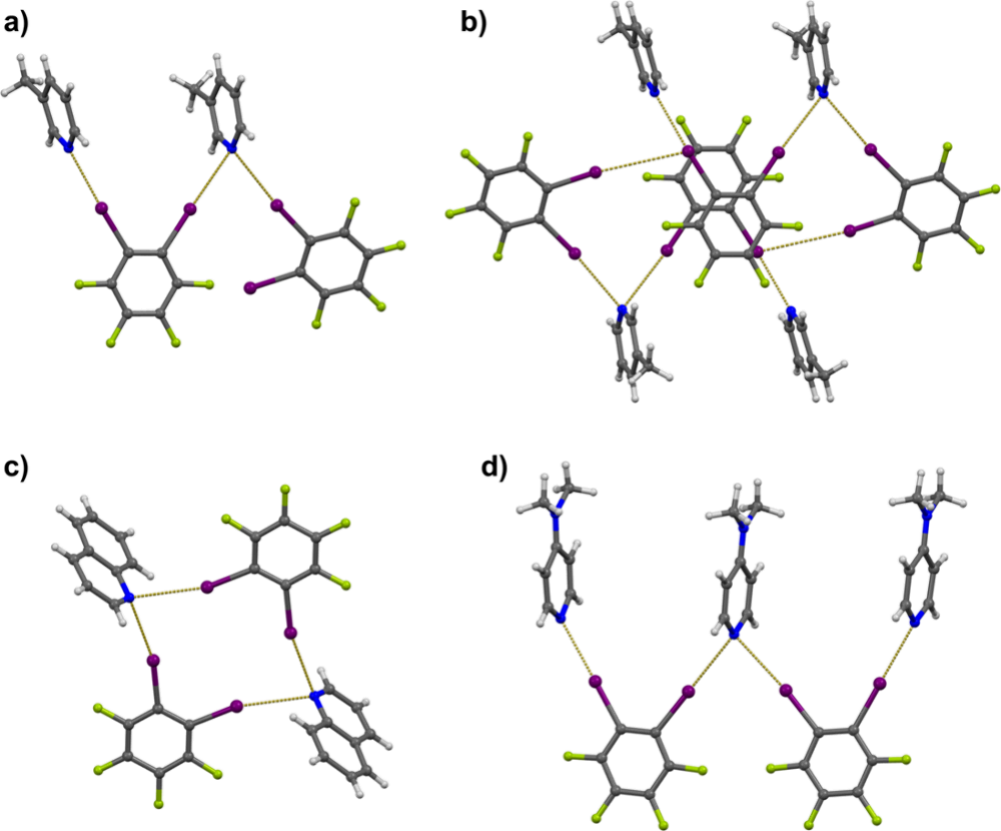
(a) Halogen-bonded tetramer in the crystal structure of (**12tfib**)­(**3pic**). (b) Halogen-bonded octamer in the crystal structure of (**12tfib**)­(**3pic**) formed by iodine···iodine interhalogen contacts between tetramers. (c) Halogen-bonded tetramer in the crystal structure of (**12tfib**)­(**quin**). (d) Halogen-bonded pentamer in the crystal structure of (**12tfib**)_2_(**dmap**)_3_·**dmap**.

Interestingly, a similar binding motif was found in the β polymorph of (**12tfib**)­(**dmap**)_2_, which was obtained by crystallization from a solution of **12tfib** and **dmap** (1:2) in a dichloromethane–hexane solvent mixture (other crystallization solvents, as well as mechanochemical syntheses, have yielded the α polymorph). Unlike the 1:2 halogen-bonded trimers present in the α polymorph, here halogen bonding leads to the formation of (**12tfib**)_2_(**dmap**)_3_ halogen-bonded pentamers in which both **12tfib** molecules act as ditopic donors, each to one terminal and one bridging **dmap** molecule, with the fourth **dmap** molecule not involved in halogen bonding, but rather binding to the central **dmap** molecule as an acceptor of a C–H···N hydrogen bond. The pyridine nitrogen of the central **dmap** molecule is again a bifurcated acceptor of two C–I···N halogen bonds with the I···N···I angle (84.6°) almost identical to that in (**12tfib**)­(**quin**). As in the previous two cases, the bifurcated I···N···I halogen bonds are somewhat asymmetric (*d*_1_ = 3.025 Å; φ_1_(C–I···N) = 173.8° and *d*_2_ = 2.965 Å; φ_2_(C–I···N) = 176.5°) as well as longer (and less linear) than I···N halogen bonds toward the terminal **dmap** molecules (*d*_1_ = 2.786 Å; φ_1_(C–I···N) = 179.7°; *d*_2_ = 2.807 Å; φ_2_(C–I···N) = 178.2°).

To get a better insight into the halogen bond donor potential of the **12tfib** molecule, we have performed DFT calculations of the molecular electrostatic potential (MEP) of the **12tfib** molecule, as well as binding energy calculations of **12tfib** with representative pyridine derivatives as halogen bond acceptors (the strongest base **dmap**, medium strong base **py**, and weak base **4cnpy**), and compared the results obtained with those for **13tfib** and **14tfib**. In this series of donors, **14tfib** exhibits the highest MEP_max_ value for donor iodine atoms at 134.5 kJ mol^–1^, followed by **13tfib** with a value of 130.8 kJ mol^–1^, while the lowest MEP_max_ value is observed in the **12tfib** molecule, which stands at 126.0 kJ mol^–1^. The difference in MEP_max_ values of **12tfib** and the remaining two perhalogenated donors is slightly less than double (6.4 kJ mol^–1^ in average) than the difference between the MEP_max_ of **13tfib** and **14tfib** molecules (3.6 kJ mol^–1^). Binding of the acceptor molecule to one of the available donor atoms of **12tfib** should further reduce the MEP_max_ value on the free donor atom, to an extent that depends on the basicity of the acceptor itself. In the specific cases investigated in this study, the greatest reduction in the MEP_max_ occurred with the most basic acceptor **dmap** (28%), followed by **py** (19%), and finally **4cnpy** (7%; Figure 4 and Table 2). Such a behavior was previously observed and quantitatively investigated in other ditopic donors **13tfib**, **14tfib** and the tritopic donor **135tfib** (Table 2). During the binding of one molecule of **4cnpy** and **py**, these donors undergo changes in MEP_max_ values similar to those observed for **12tfib**.^39^ However, in the case of **12tfib**, the effect of the basicity of the acceptor is more pronounced than in the case of its congeners, as binding of the least basic acceptor (**4cnpy**) affects the MEP_max_ value on the free donor atom of other studied donors somewhat more, whereas the most basic (**dmap**) has a considerably smaller effect on the other donors than on **12tfib**. The reduced MEP_max_ on the free iodine atom also causes a decrease in the binding energy of the second acceptor molecule with respect to the previously formed 1:1 (**donor**)­(**acceptor**) complex. The trend of decreasing the second binding energy is the same as for donors **13tfib**, **14tfib**, and **135fib**: the largest reduction of the binding energy is for **dmap**, followed by **py**, and finally, **4cnpy**. In spite of this reduction, the binding of the stronger bases remains more favorable than binding of the weakly basic **4cnpy**, which accounts for the formation of a 1:2 cocrystal with **dmap**, while no cocrystal with **4cnpy** could be obtained.

**Figure 4:**
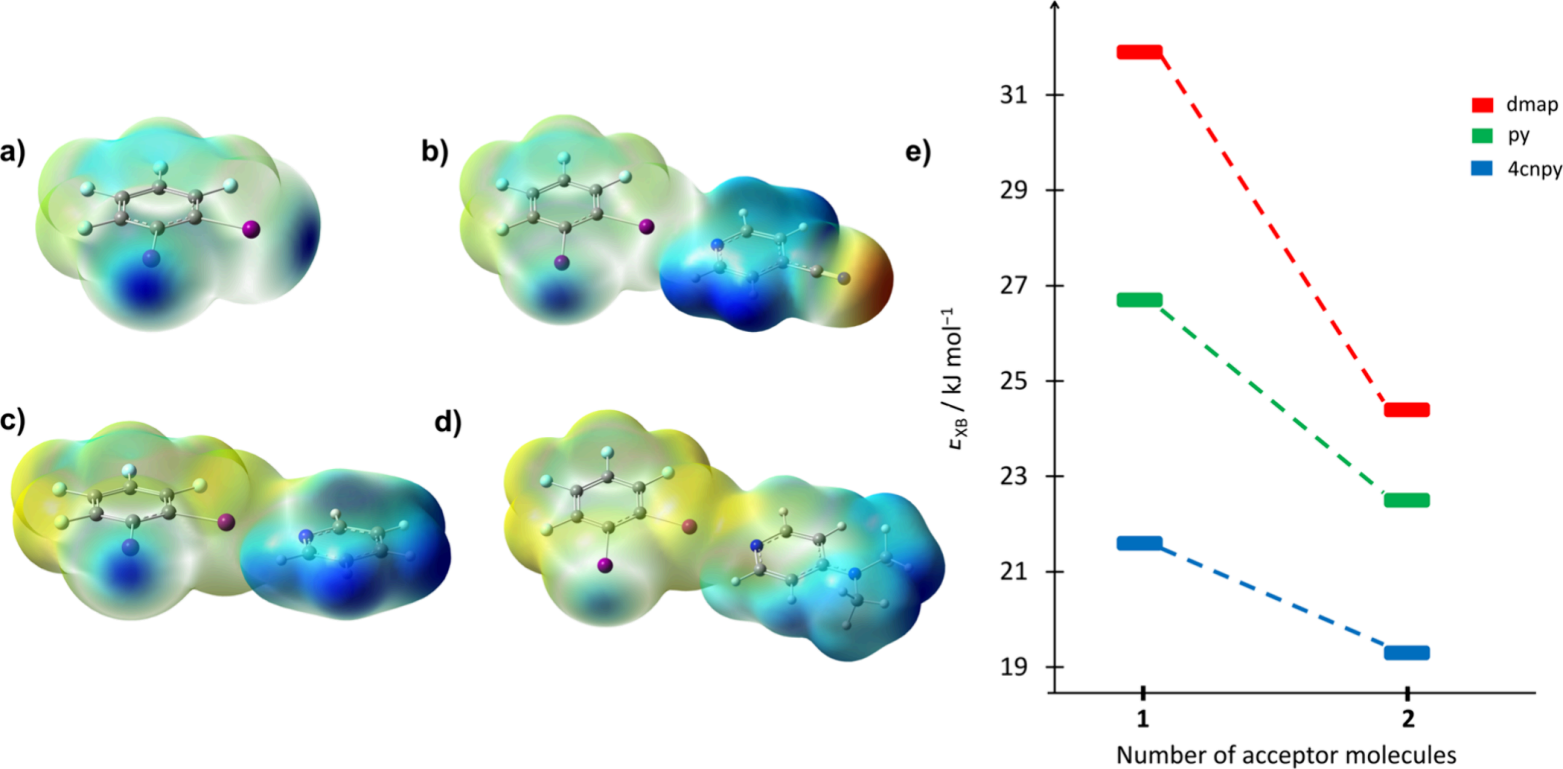
Molecular electrostatic potential mapped on the electron isodensity surface (0.001 a.u.) of a)**12tfib**, b) (**12tfib**)­(**4cnpy**) molecular complex, c) (**12tfib**)­(**py**) molecular complex, d)­(**12tfib**)­(**dmap**) molecular complex, and e) first and second binding energies of **dmap**, **py**, and **4cnpy**to **12tfib**.

**Table 2:** Comparison of the Reduction of MEP_max_ Values Plotted on the 0.001 a.u. Electron Isodensity Surface (% of the MEP_max_ on the Free Donor Molecule) on the Free Iodine Atoms of **12tfib**, **13tfib**, **14tfib**, and **135tfib** Upon Binding of One Acceptor Molecule

donor	**4cnpy**	**py**	**dmap**
**12tfib**	7.2	19.0	28.5
**13tfib**	9.1	16.7	24.2
**14tfib**	10.1	18.8	25.4
**135tfib**	10.2	18.8	25.8

The fact that in two obtained crystal structures the nitrogen atom appeared as a bifurcated acceptor of a pair of halogen bonds prompted us to perform a series of computations of binding energies in such systems in order to ascertain whether there is an advantage in forming such (unusual) halogen bonds or their appearance might simply be due to specific packing effects in these structures. For this purpose, we have compared the energy of halogen bonding involved in the formation of a (**12tfib**)_2_(**quin**)_2_ tetramer in the gas phase, with that which would be involved in forming a *classical* (**12tfib**)­(**quin**) dimer (with only a single halogen bond). The DFT calculation has shown the (**12tfib**)_2_(**quin**)_2_ tetramer to be a stable configuration in the gas phase, with the optimized configuration similar to that found in the crystal structure, and the total energy for formation of a (**12tfib**)_2_(**quin**)_2_ tetramer from two **12tfib** and two **quin** molecules is 83.43 kJ mol^–1^. Since the formation of the tetramer involves the formation of four halogen bonds with nitrogen as the bifurcated acceptor, this corresponds to an average halogen bond energy of 20.86 kJ mol^–1^. When this is compared to the halogen bond in the (**12tfib**)­(**quin**) dimer of 27.28 kJ mol^–1^, it is immediately obvious that the linear halogen bond of a nearly optimal geometry in the (**12tfib**)­(**quin**) dimer (with C–I donor group approximately in the plane of the quinoline molecule) is by ca. 6.5 kJ mol^–1^ more favorable than the halogen bond with a bifurcated nitrogen (where the C–I donor group approaches the quinoline plane at an angle of 171°). However, as the formation of a (**12tfib**)_2_(**quin**)_2_ tetramer involves the formation of four halogen bonds, whereas in the (**12tfib**)­(**quin**) dimer there is only one, the tetramer is in fact more stable than a pair of dimers by 28.87 kJ mol^–1^. In order to ascertain whether this is a unique feature of quinoline as an acceptor, an identical set of calculations was performed with pyridine as the acceptor. While these computations, expectedly, did result in different values of the halogen bond energies, the difference between the energies of the halogen bonds in the (**12tfib**)­(**py**) dimer and the (**12tfib**)_2_(**py**)_2_ tetramer is similar to that in (**12tfib**)_2_(**quin**)_2_ (19.24 kJ mol^–1^ per bond in the tetramer and 27.28 kJ mol^–1^ in the dimer–difference of ca. 7.5 kJ mol^–1^), as well as the general trend of the tetramer being more stable (by 23.57 kJ mol^–1^) than a pair of dimers. However, when the strongly basic **dmap** was introduced as the halogen bond acceptor, an equivalent (**12tfib**)_2_(**dmap**)_2_ tetramer did no longer correspond to an energy minimum, and the optimization of the geometry has yielded a pair of (**12tfib**)­(**dmap**) monomers exhibiting linear halogen bonds. Similarly, geometry optimization of the (**12tfib**)_2_(**dmap**)_3_ pentamer resulted in the formation of the (**12tfib**)­(**dmap**)_2_ trimer and the (**12tfib**)­(**dmap**) monomer. It appears therefore that with weaker bases as halogen bond acceptors, the tetramer comprising four halogen bonds with nitrogen as a bifurcated acceptor is generally a more favorable configuration than a pair of dimers. The absence of the tetramers in the crystal structures, rather than their presence, is a result of crystal packing.

Of further interest was to examine this angular dependence in systems with a pair of C–I halogen bond donor groups in a configuration suitable for achieving bifurcated binding to the pyridine nitrogen. This was examined in three model systems: one involving a pyridine molecule and two iodopentafluorobenzene molecules as simple model C–I halogen bond donors, one with a pyridine molecule and a pair of **12tfib** molecules as donors, and finally, a (**12tfib**)_2_(**py**)_2_ tetramer. In each case, the C–I halogen bond donor groups were held in constant positions, and the pyridine ring was placed symmetrically between them (both C–I bonds at an angle of 132.7° to the pyridine ring) and then tilted to a highly asymmetric configuration (174.6° toward one and 87.7° toward the other C–I bond) with 0.2–0.7° increments. Interestingly, while the absolute energies differ in the three mode systems, the overall shapes of the curves, as well as their slopes at corresponding values of the angle, are almost identical. The energy curve indicates a wide single-well potential with only a slight indication of an energy barrier (the energy of the perfectly symmetric configuration merely ca. 0.01 kJ mol^–1^ higher than that tilted by 0.2°). The angular dependence of the energy is also rather low, less than 5 kJ mol^–1^ over the entire studied region. If this curve is compared to the sum of the effects of two independent C–I donors (Figure 5), it can be seen that they correspond in the position of the minimum; however, the energy minimum of the actual energy curve is ca. 5 kJ mol^–1^ higher than the sum of the contributions of two independent C–I donors, and the latter curve exhibits a considerably steeper slope. The overall shape of the energy curve for a dual C–I donor site can be explained by the two donor groups compensating for each other: as the angle of one C–I bond decreases from the most favorable angle, thus decreasing its contribution to the overall interaction energy, the angle of the other increases, making its contribution to the interaction energy larger. Therefore, it would appear that while the symmetrical (or approximately symmetrical) configuration of a pair of halogen bonds formed with the same pyridine nitrogen atom, in the solid state one might expect considerable deviations from this geometry, as the required energies are easily attainable through crystal packing effects.

**Figure 5:**
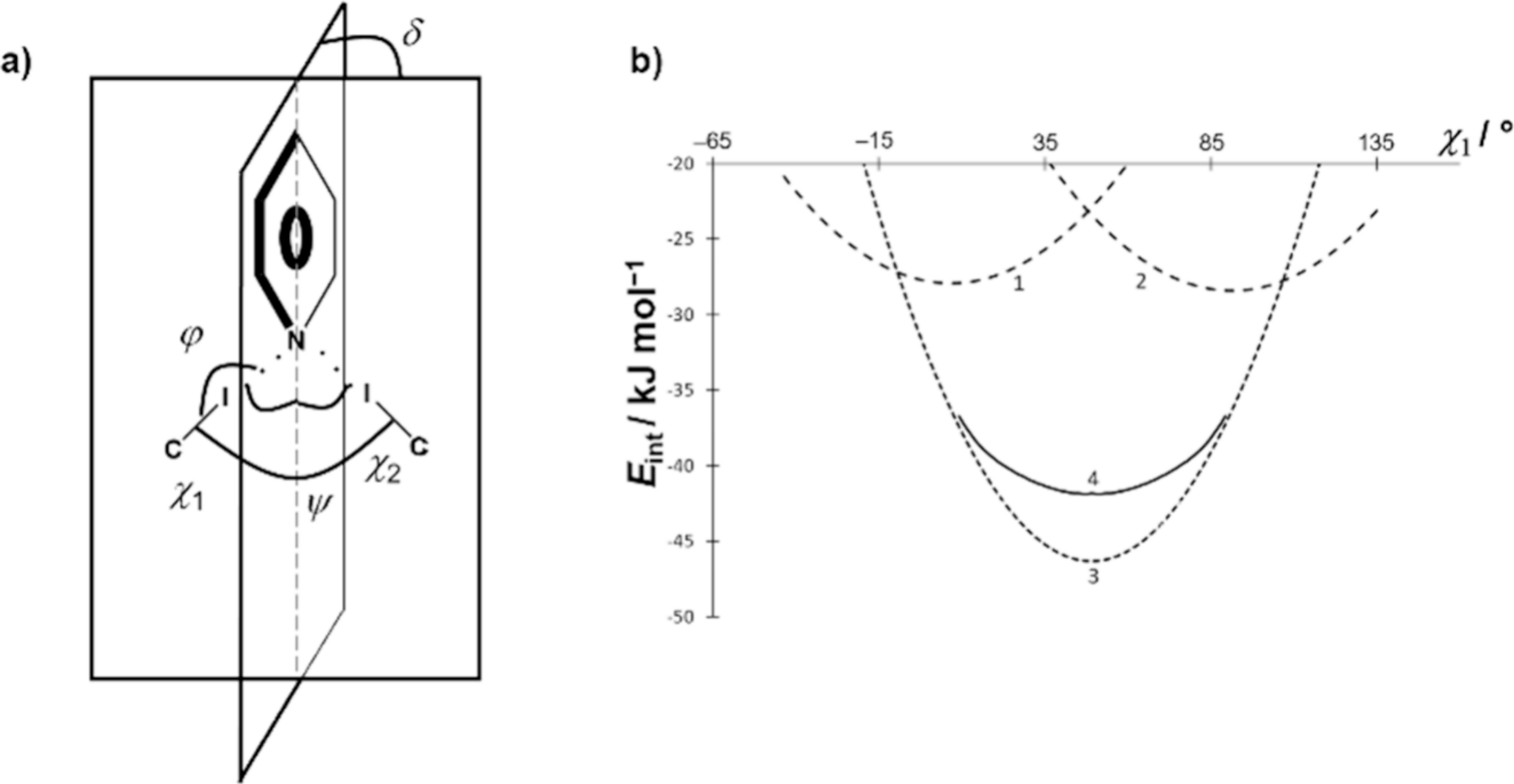
(a) Definition of angles relevant for the description of the halogen bonds with pyridine nitrogen as a bifurcated acceptor of two halogen bonds: φ = halogen bond angle, χ_1_, χ_2_ = the angles of approach of the two C–I bonds to the plane of the sp^2^ system, ψ = the angle between the vectors of the C–I bonds, and δ = dihedral angle between the plane of the sp^2^ system and the plane defined by the acceptor nitrogen and the donor iodine atoms. (b) Angular dependence of halogen bond potential energy as a function of χ1 (for δ = 90°, ψ = 90°, *d*(I···N) = 3.045 Å): 1: if only one acceptor molecule is present, 2: if only second acceptor molecule is present, 3: sum of 1 and 2, and 4: actual potential energy curve when both acceptors are present.

The occurrence of a bifurcated nitrogen acceptor has prompted us to perform a more detailed CSD search to investigate whether this phenomenon can be observed more generally. The search was constrained to (tertiary) sp^2^ nitrogen atoms as halogen bond acceptors and C–I halogen bond donors. For 1501 entries, a C–I···N halogen bond was found. Of these, only in 36 entries, the nitrogen atom was forming two contacts with iodine atoms, of which 3 were found to comprise only a single halogen bond C–I···N halogen bond and an incidental I···N contact, leaving 33 data sets (ca. 2.2% of the total) where the nitrogen atom was a bifurcated acceptor, indicating that this is an extremely rare occurrence. It is noteworthy that of these 33 entries, over one-third (12) correspond to structures comprising halogen bond donors with vicinal (i.e., *ortho*) iodine atoms (7 structures with **12tfib** and 5 with other *ortho*-diiodo species: tetraiodoethene and complexes with iodinated ligands). Furthermore, as there were only 124 entries comprising any halogen bonding between an *ortho*-diiodo species and a tertiary sp^2^ nitrogen, the occurrence of nitrogen acting as a bifurcated acceptor within this group is ca. 10%, considerably higher than the general population. This trend is continued when the search is constrained to perfluorinated iodobenzenes in cocrystals with N­(sp^2^)-heterocycles: **135tfib** forms 7 cocrystals with bifurcated N­(sp^2^) (of overall 97 with N­(sp^2^), i.e., ca. 7%), **14tfib** forms 6 (out of 300; 2%), **13tfib** forms none (out of 45), and **12tfib** forms 7 (out of 47; 15%). Overall, cocrystals of **12tfib** account for 35% of structures with a bifurcated N­(sp^2^) acceptor, although they account for less than 10% of the overall number of cocrystals of perfluorinated iodobenzenes with N­(sp^2^)-acceptors. It is also noteworthy that 5 out of 7 **12tfib** cocrystals with bifurcated N­(sp^2^) acceptors comprise the (**12tfib**)_2_(**A**)_2_ tetramers, while only a single other similar tetramer with another donor (VIXXAE with **135tfib**) was found in the database search.

As it could be expected, when a sp^2^ nitrogen atom acts as an acceptor for two halogen bonds, they are on average somewhat longer (3.22 ± 0.23 Å) and less linear (160 ± 18°) than in the cases when only a single halogen bond is present (average bond length for sp^2^ nitrogen acceptors and C–I donors is 2.982 ± 0.20 Å and angle 170 ± 13°). There does not seem to be any statistically significant difference between the cases the donor is or is not an *ortho*-diiodo species, either in the average bond lengths (3.20 ± 0.23 Å, vs 3.23 ± 0.24 Å) or in the average bond angles (159 ± 14°, vs 161 ± 20°), although the deviation from the average value is significantly smaller among the structures comprising *ortho*-diiodo halogen bond donors. This closer grouping of data points is even more pronounced when inspecting the halogen bond length vs angle scatterplot (Figure 6a). There is a fairly narrow band following the generally diagonally descending trend (longer halogen bonds are also less linear), which contains the vast majority of the data points corresponding to halogen bonds with ortho-diiodo halogen bond donors. The only significant outliers are bifurcated halogen bonds in two 1,10-phenanthroline structures (CSD refcodes QEFNAU and YANPIP) where the iodine is in contact with both nitrogen atoms in the acceptor molecule. Another rare outlier is PERLEG, in which there is a dominant (linear and short) halogen bond with a donor lying close to the sp^2^ plane and a secondary (considerably longer) contact approaching the plane at a high angle (also see discussion below).

**Figure 6:**
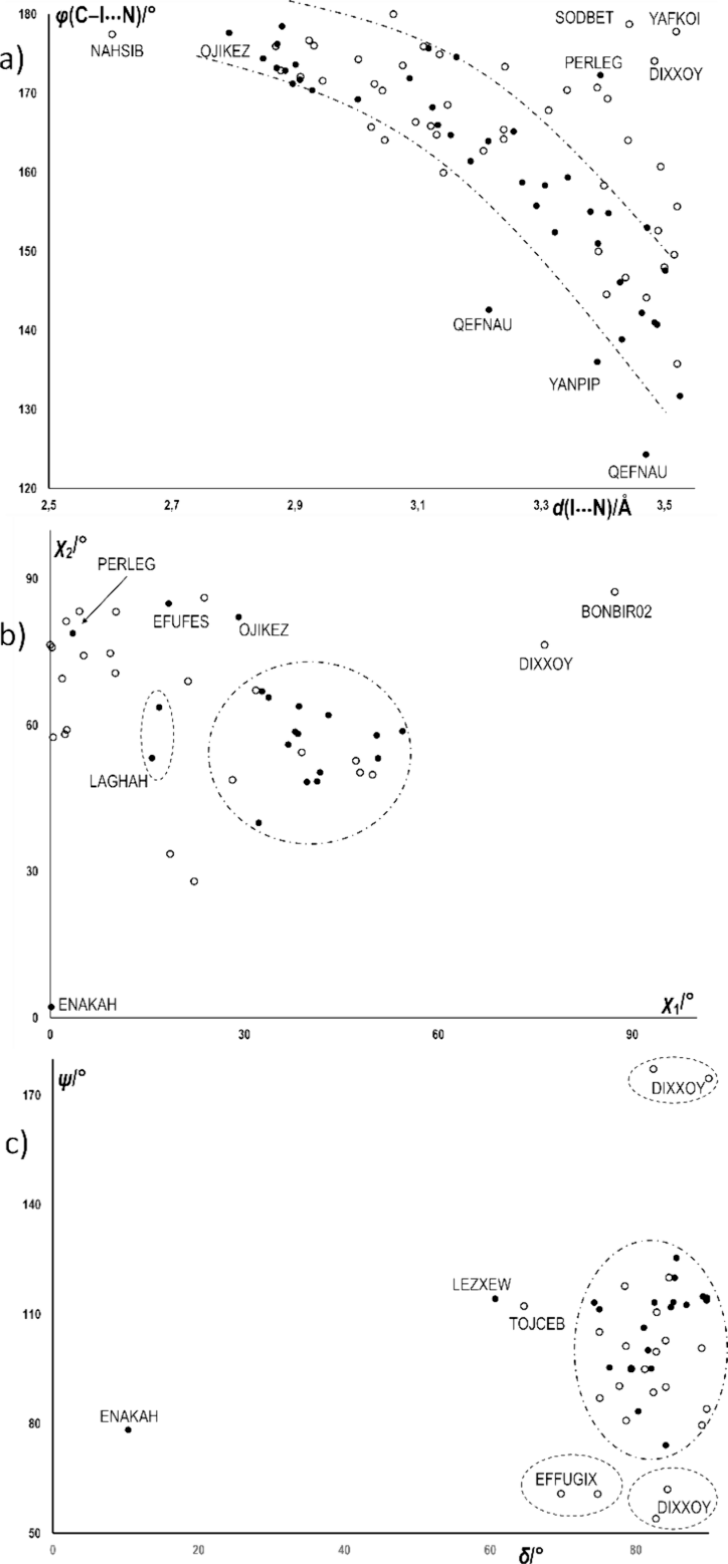
Geometric parameters for halogen bonding with the bifurcated N­(sp^2^) acceptor. (a) Halogen bond length vs angle scatterplot; (b) the angles of approach of the two C–I bonds to the plane of the sp^2^ system (for the simplicity of representation, always defined such that χ_1_ ≤ χ_2_); and (c) dihedral angle between the plane of the sp^2^ system and the plane defined by the acceptor nitrogen and the donor iodine atoms (δ) vs the angle between the vectors of the C–I bonds (ψ). The full circles correspond to structures comprising *ortho*-diiodo halogen bond donors and the dot-dash lines enclose the area where the majority of the data points corresponding to the bonds with *ortho*-diiodo halogen bond donors are concentrated. The denoted CSD refcodes correspond to outliers, which are discussed in the text.

Generally, the angles of approach for the two C–I donor groups to the plane of the sp^2^-N acceptor (defined as the plane containing the sp^2^-nitrogen atom and its two immediate neighbors, which approximately coincides with the mean molecular plane for aromatic heterocycles) are between ca. 30 and 90°, albeit dispersed over a large range (an average value of 48° with a standard deviation of 27°), particularly for structures where the donors are not ortho-diiodo donors (51 ± 31°), while the distribution is considerably narrower in the case of *ortho*-diiodo donors (45 ± 20°). The origin of this difference becomes clear when the scatterplot of the two angles is observed (Figure 6b). In the majority of the structures of the *ortho*-diiodo donors, one C–I donor group approaches the sp^2^-N plane at 30–55° from one side of the plane, while the other one approaches at 40–70° from the other side, resulting in the data points accruing close to the middle of the plot. This indicates a general proclivity of the *ortho*-diiodo halogen bond donors toward approximately symmetrical coordination of the donors to the acceptor nitrogen. On the other hand, while there are some data points corresponding to the structures of non-*ortho*-diiodo species in this region, the majority are on the top left, corresponding to coexistence of one almost linear contact (with a low χ_1_ angle) and the other donor approaching at a high angle, with the difference between the two generally larger than 60° (e.g., NAHSIB, YAFKOI, EFFUGIX, DIXXOY, SODBET), indicating a tendency toward highly asymmetric binding. However, such a binding motif is very rare among the structures comprising *ortho*-diiodo donors and is found in only four examples (OJIKEZ, EFUFES, LAGHAH, and the beforementioned PERLEG).

It should be noted that in both groups of structures there are extreme outliers, albeit few and specific. Among the structures comprising *ortho*-diiodo donors, this is ENAKAH, a diiodotriazole in which both C–I donors form somewhat bifurcated bonds, one with a pair of contiguous nitrogen atoms, the other with a nitrogen and an iodine from a third molecule, leading to an approximately coplanar arrangement of the molecules and therefore both χ_1_ and χ_2_ close to 0°. This unique geometric feature is also illustrated by the dihedral angle between the plane of the sp^2^ system and the plane defined by the acceptor nitrogen and the donor iodine atoms of ca. 10°, while in all other structures, this angle is above 60° (and generally above 75°) (Figure 6c). The other extreme is represented by DIXXOY and BONBIR02 (both with non-*ortho*-diiodo donors), where both angles approach 90°. Of these, the former represents a unique case where a pyridine nitrogen forms three halogen-bonding contacts, one approximately in-plane (13.58°) and two approximately perpendicular to it at opposite sides.

In spite of a wide variation in approach angles χ_1_ and χ_2_, the angle between the two approaching C–I bonds (ψ) is much more constant among the data set (Figure 6 With the exception of DIXXOY where there are C–I donors on opposite sides of the acceptor (i.e., at ca. 180°), all values of the ψ angle fall within the 55–125° range, and the majority spaced within the more narrow 75–125° range. There is apparently a slight tendency that the less symmetrical arrangements of the C–I donors are connected with lower ψ angles, which is generally to be expected since the most negative region of the acceptor is in the plane of the sp^2^ system, and therefore, the C–I donors at high χ_2_ angles are approaching the acceptor from a less favorable direction. On the other hand, the minimum ψ angle is determined by the radius of the iodine. Indeed, in all cases with ψ angle below 75°, the nonbonded I···I distance is within 1.5% of the double van der Waals radius of iodine (3,96 Å).

## Conclusions

Generally, the relatively low number of published cocrystal structures with **12tfib** as a donor can be only partially attributed to intrinsic factors. It does have the lowest MEP_max_ values corresponding to the iodine σ-hole when compared to its isomers, but this is less than 5% lower than the highest value (in **14tfib**). Also, mechanochemical cocrystal screening with the selected 14 bases has yielded new phases in 11 combinations, which is more than that had previously been obtained with **13tfib** (9) using the same set of nitrogen bases, albeit less than with **14tfib** (13) or **135tfib** (12).

When cocrystallized with nitrogen bases, **12tfib** generally follows the trend of forming cocrystals with a larger number of base molecules when stronger bases are employed more closely than the 1,3 and 1,4 isomers. With weak bases (p*K*_a_ < 4), no cocrystals were obtained; with intermediate bases (4 < p*K*_a_ < 6.2), it formed cocrystals predominantly of 1:1 stoichiometry, while the strongest two bases (p*K*_a_ > 6.2) yielded cocrystals of 1:2 stoichiometry. This is in line with the computed binding energies of one and two base molecules on **12tfib**. Although the energy for binding of the second base molecule is much more affected by the base strength than in the case of other perfluorinated aromatic donors, the energy for binding the second molecule of a strong base (**dmap**) is still greater than that for binding the first molecule of a weak base (**4cnpy**), thus rationalizing the observed tendencies for **12tfib** to form cocrystals only with stronger bases.

The less expected observation, however, was the tendency of the N­(sp^2^) atom to act as a bifurcated acceptor with **12tfib** as a halogen bond donor. This behavior, observed in three herein presented cocrystals and shown to produce stable oligomers in vacuo, has been confirmed by CSD data mining to be statistically most likely not only for **12tfib** as a halogen bond donor but for other *ortho*-diiodo species as well. While the currently available structural and computational data did allow drawing some conclusions on the occurrence, geometries, and energetics of halogen bonding involving bifurcated sp^2^ nitrogen acceptors, additional studies of this phenomenon will be necessary.

## Experimental Section

### Crystallization Experiments

Single crystals suitable for SCXRD experiments were prepared by slow evaporation of 3.0 mL of the ethanolic solution of **12tfib** (0.50 mmol) and the corresponding pyridine base in a 1:2.5 molar ratio (to ensure excess of the acceptor) at ca. 25 °C. Single crystals formed within 1 to 2 days.

### Mechanochemical Experiments

The neat grinding experiments were conducted in a Retsch MM200 ball mill using 10 mL stainless steel jars and two stainless steel balls (5 mm in diameter) for 15 min at 25 Hz, under normal laboratory conditions (40–60% relative humidity and temperature ca. 25 °C). It has been observed that such experimental conditions are sufficient for the synthesis of cocrystals containing perhalogenated donors and simple pyridines, and often longer grinding times have no effect on the formation of the desired product or may lead to amorphization of the reaction mixture. Masses and volumes of the reactants used in the mechanochemical synthesis of cocrystals are given in Table S1 in the Supporting Information.

### Single-Crystal X-ray Diffraction Experiments

The crystal and molecular structures of the prepared cocrystals were determined by single-crystal X-ray diffraction. Diffraction measurements were made on a Rigaku Synergy XtaLAB X-ray diffractometer with graphite-monochromated MoKα (λ = 0.71073 Å) radiation. The data sets were collected using the ω scan mode over the 2θ range up to 64°. Programs CrysAlis CCD, CrysAlis RED, and CrysAlisPro were employed for data collection, cell refinement, and data reduction.^46,47^ The structures were solved by direct methods and refined using the SHELXS and SHELXL programs.^48,49^ The structural refinement was performed on *F* ^2^ using all of the data. Hydrogen atoms were placed in calculated positions and treated as riding on their parent atoms. All calculations were performed using the WINGX crystallographic suite of programs.^50^ The molecular structures of compounds and their molecular packing projections were prepared by Mercury.^51^

### Powder X-ray Diffraction Experiments

PXRD experiments were performed on a Malvern PANalytical Aeris X-ray diffractometer with CuKα1 (1.54056 Å) radiation at 15 mA and 40 kV. The scattered intensities were measured with a scintillation counter. The angular range was from 5 to 40° (2θ) with steps of 0.02–0.03°, and the measuring time was 0.2–0.5 s per step. Data collection and analysis were performed using the program packages Data Viewer^52^ and High Score.^53^

### Calculation Details

All calculations were performed using the Gaussian 16 software package.^54^ Geometry optimizations were performed using the M062X/def2-tzvp level of theory,^55^ with an ultrafine integration grid (99 radial shells and 590 points per shell). It has been shown that this functional in combination with the triple-ζ basis set provides quite accurate geometries of halogen-bonded molecular complexes as well as their energies.^56,57^ The same level of theory was used to calculate the binding energies on optimized geometries, employing the Boys–Bernardi counterpoise scheme^57^ to account for the basis set superposition error. Harmonic frequency calculations were performed on the optimized geometries to ensure the success of each geometry optimization. The figures were prepared using GaussView.^58^

Repulsion energies between two bonded acceptor molecules in (**12tfib**)­(**acceptor**)_2_ molecular complexes were calculated in Crystal Explorer^59^ using the b3lyp/6-31g­(d,p) level of theory^60^.

## Supplementary Material



## Notes

Comparison of the XRPD patterns of reaction mixtures and calculated XRPD patterns of the prepared cocrystals; masses and volumes of reactants used in grinding experiments; halogen bond lengths and angles in the prepared cocrystals; optimized structures of supramolecular dimers, trimers, and tetramers of 12tfib with **py**, **quin**, and **dmap**; parameters of halogen bonds with the nitrogen atom as a bifurcated acceptor; Cartesian XYZ coordinates of the optimized geometries; and molecular electrostatic potential maps of py, quin, and dmap
