# Supplementary material for: Cocrystals of 1,2-Diiodotetrafluorobenzene with Pyridine Derivatives: Pyridine Nitrogen as a Bifurcated Acceptor with Ortho-Diiodo Halogen Bond Donors
Source: Cryst Growth Des. 2025 Dec 23;26(1):554–64. doi: 10.1021/acs.cgd.5c01439 (PMC12787666; doi:10.1021/acs.cgd.5c01439)
Supplement: Supplementary file 1 [file cg5c01439_si_001.pdf]

Cocrystals of 1,2-Diiodotetrafluorobenzene with pyridine derivatives – pyridine nitrogen as a bifurcated acceptor with ortho-diiodo Halogen Bond Donor

Nikola Bedeković, Antonio Magnabosco, Vladimir Stilinović, Dominik Cinčić

[1]Department of Chemistry, Faculty of Science, University of Zagreb, Horvatovac 102a Zagreb, Croatia.

E-mail: dominik@chem.pmf.hr, vstilinovic@chem.pmf.hr

SUPPORTING INFORMATION

Table of contents

Comparison of the XRPD patterns of reaction mixtures and calculated XRPD patterns of the prepared cocrystals (pages 2–8)

Masses and volumes of reactants used in grinding experiments (page 9)

Halogen bond lengths and angles in the prepared cocrystals (page 10)

Optimized structures of supramolecular dimers, trimers and tetramers of 12tfib with **py**, **quin** and **dmap** (page 11–13)

Parameters of halogen bonds with nitrogen atom as a bifurcated acceptor (page 14–15)

Cartesian XYZ coordinates of the optimized geometries (page 16–35)

Molecular electrostatic potential maps of py, quin and dmap (page 36)

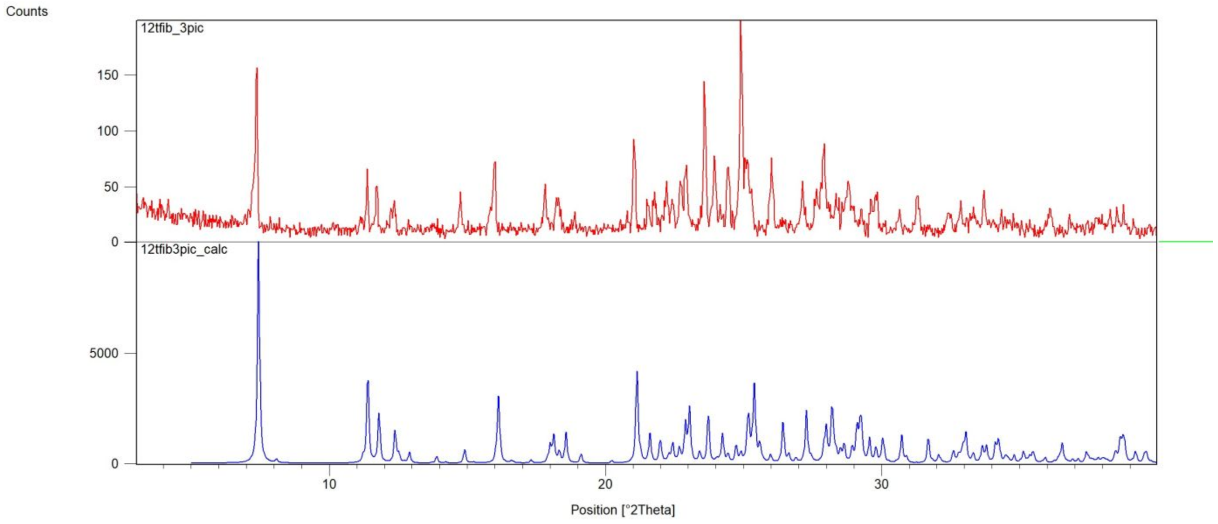

Figure S1. Comparison of the experimental XRPD pattern of the reaction mixture after grinding and calculated XRPD pattern of the (**12tfib**)(**3pic**).

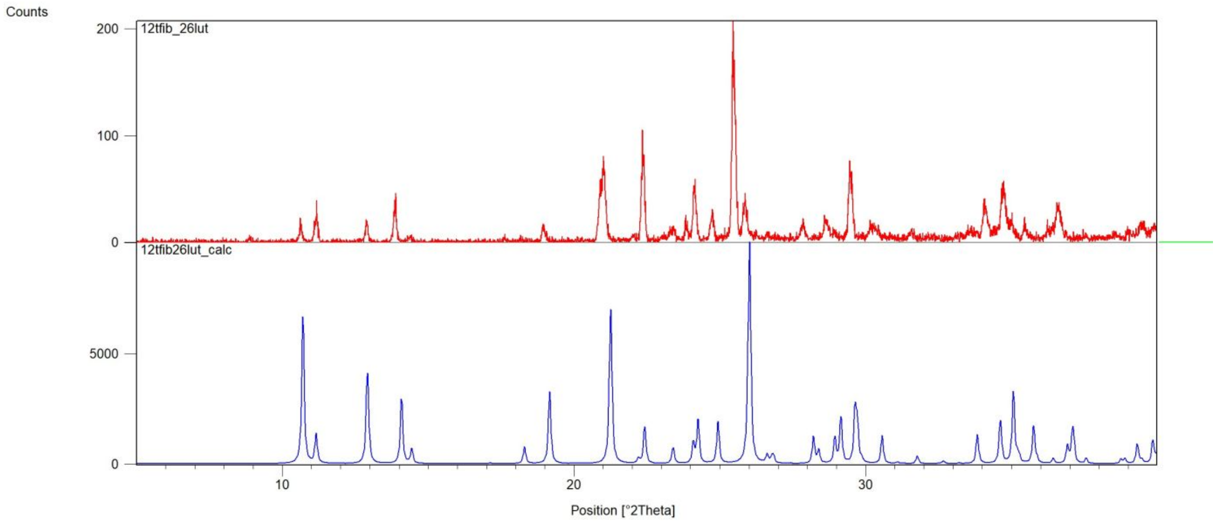

Figure S2. Comparison of the experimental XRPD pattern of the reaction mixture after grinding and calculated XRPD pattern of the (**12tfib**)(**26lut**).

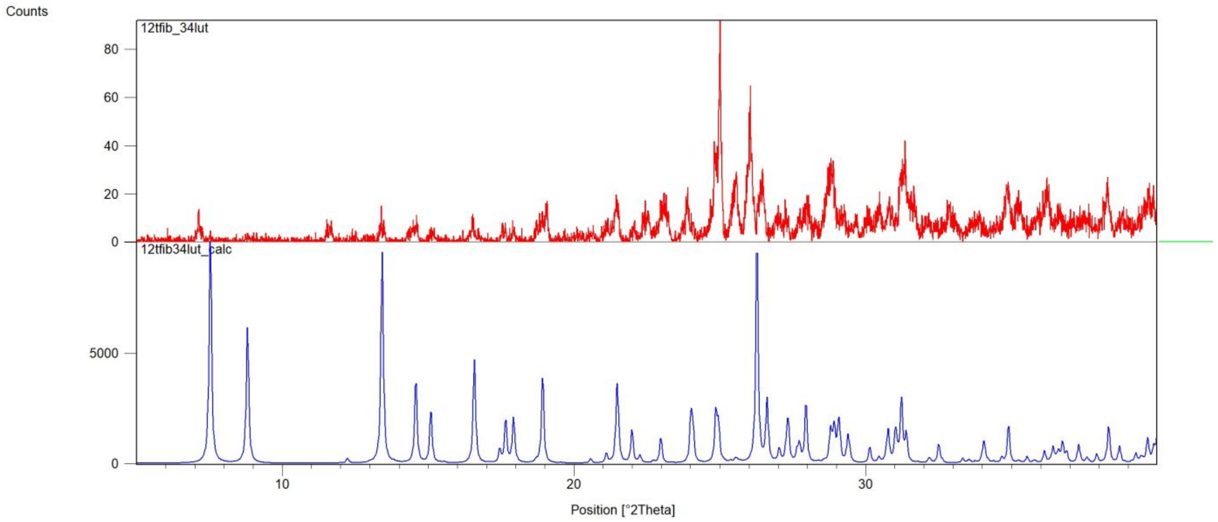

Figure S3. Comparison of the experimental XRPD pattern of the reaction mixture after grinding and calculated XRPD pattern of the (**12tfib**)(**34lut**)$_2$.

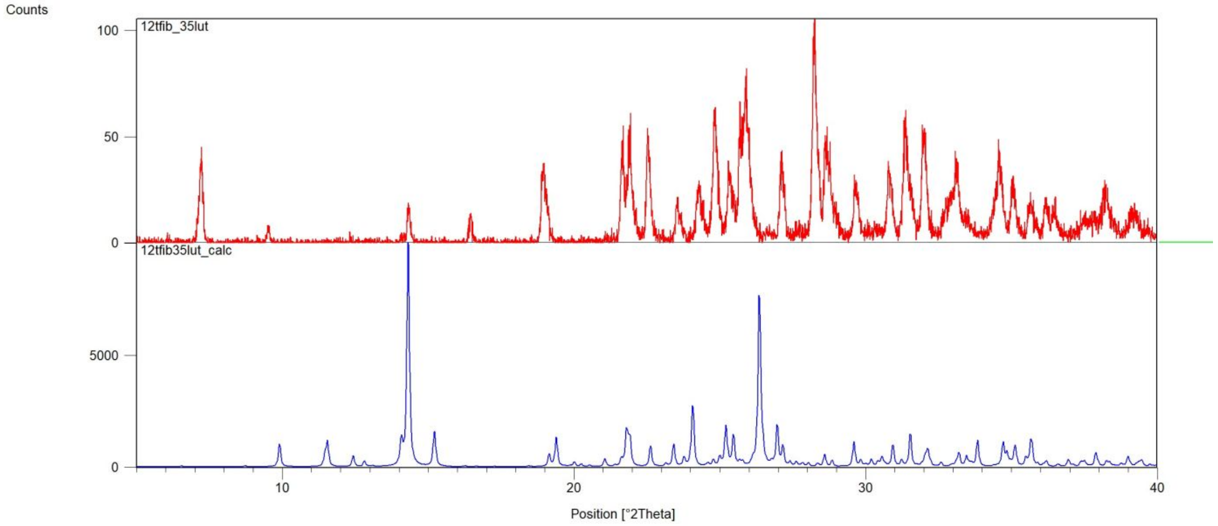

Figure S4. Comparison of the experimental XRPD pattern of the reaction mixture after grinding and calculated XRPD pattern of the (**12tfib**)(**35lut**).

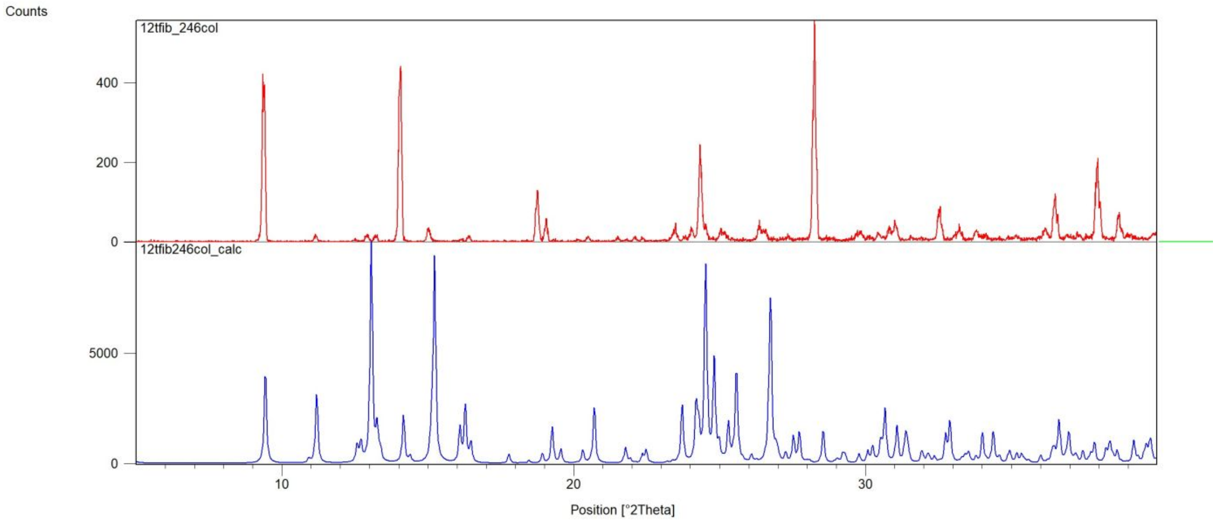

Figure S5. Comparison of the experimental XRPD pattern of the reaction mixture after grinding and calculated XRPD pattern of the (**12tfib**)(**246col**)$_2$.

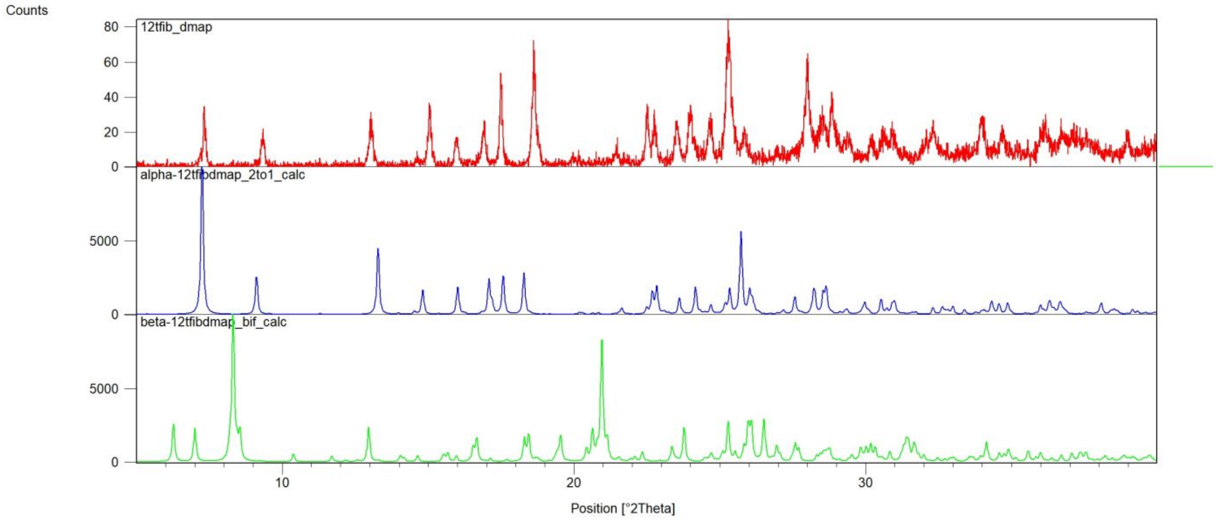

Figure S6. Comparison of the experimental XRPD pattern of the reaction mixture after grinding and calculated XRPD patterns of the (**12tfib**)(**dmap**)$_2$ and (**12tfib**)$_2$(**dmap**)$_3$(**dmap**).

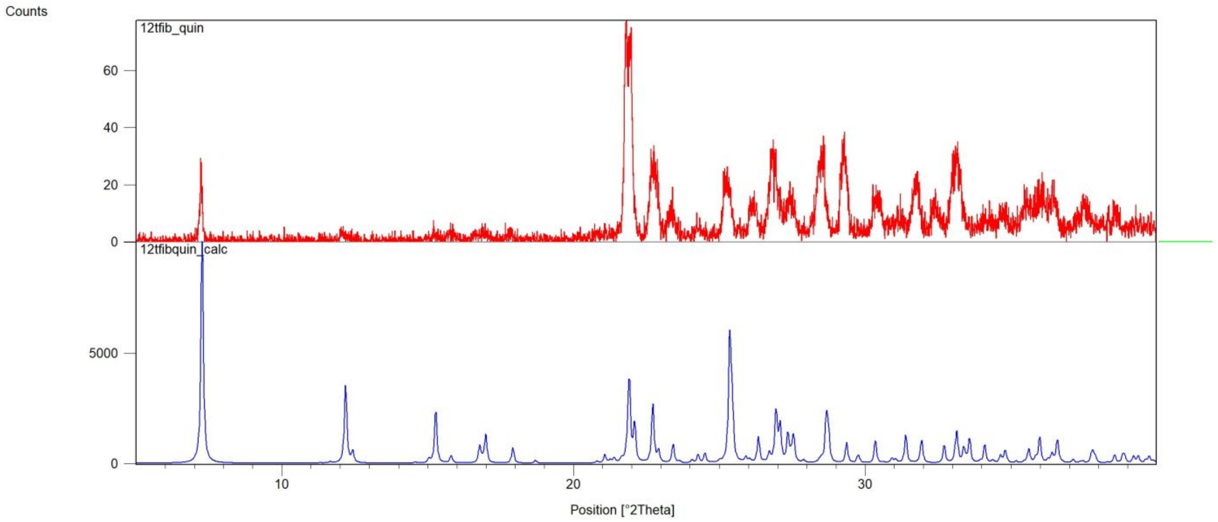

Figure S7. Comparison of the experimental XRPD pattern of the reaction mixture after grinding and calculated XRPD pattern of the (**12tfib**)(**quin**).

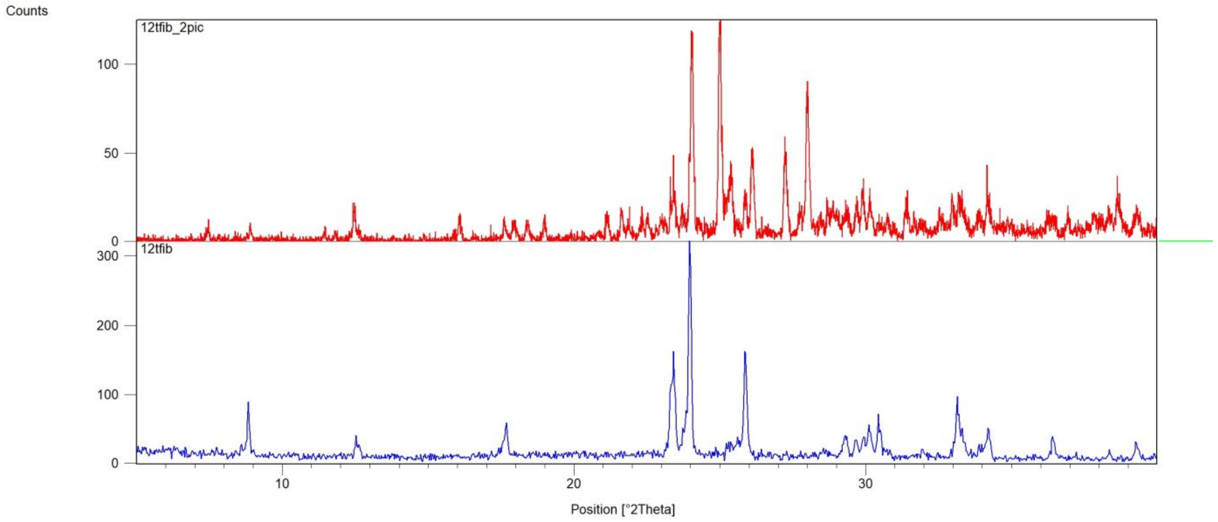

Figure S8. Comparison of the experimental XRPD pattern of the reaction mixture (**12tfib** + **2pic**) after grinding and XRPD pattern of the (**12tfib**).

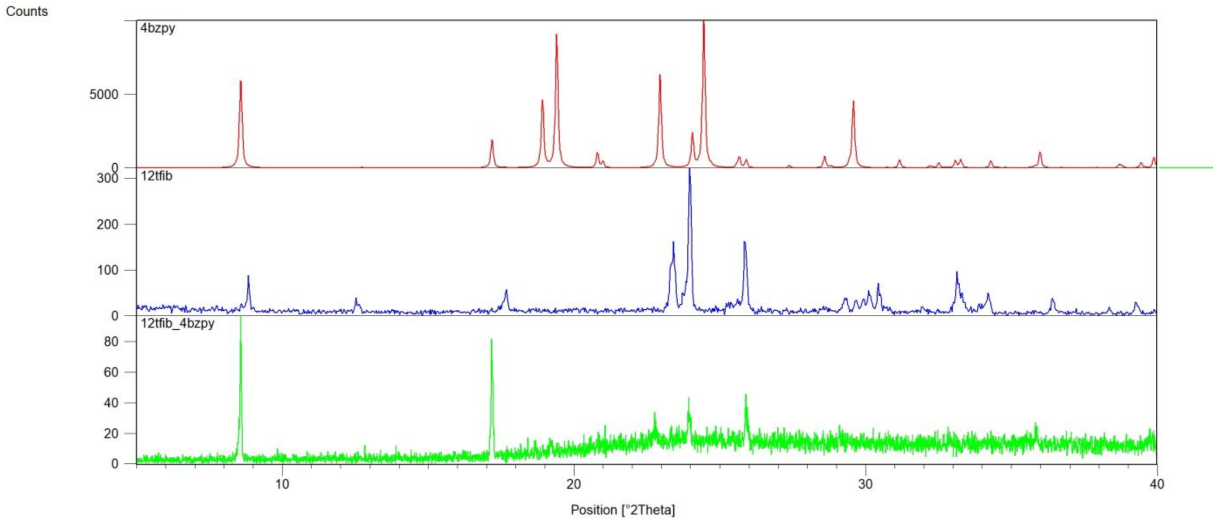

Figure S9. Comparison of the experimental XRPD pattern of the reaction mixture (**12tfib** + **4bzpy**) after grinding and XRPD pattern of the (**12tfib**).

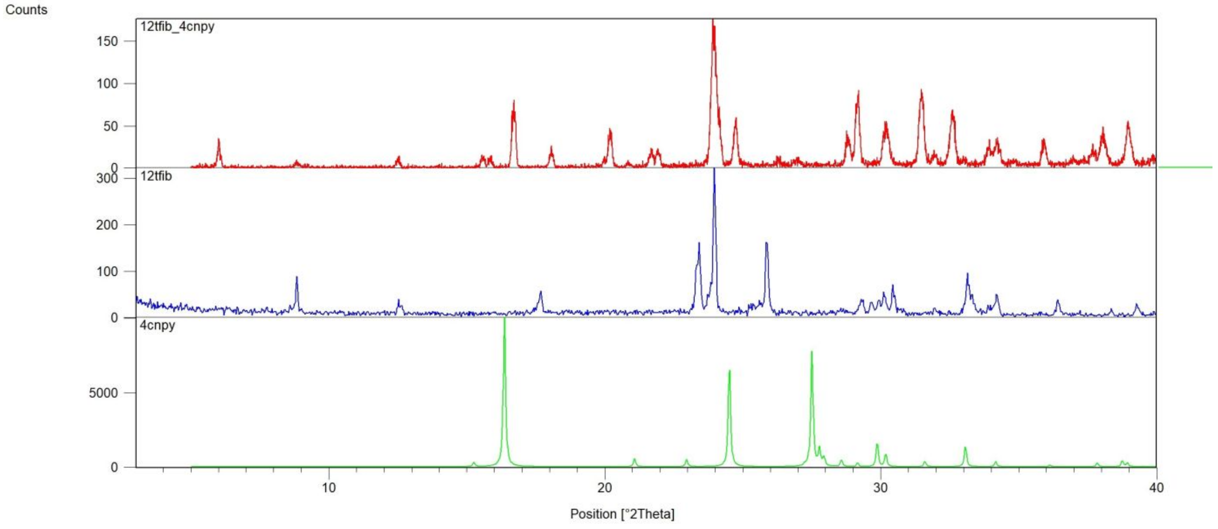

Figure S10. Comparison of the experimental XRPD pattern of the reaction mixture (**12tfib** + **4cnpy**) after grinding and XRPD pattern of the (**12tfib**).

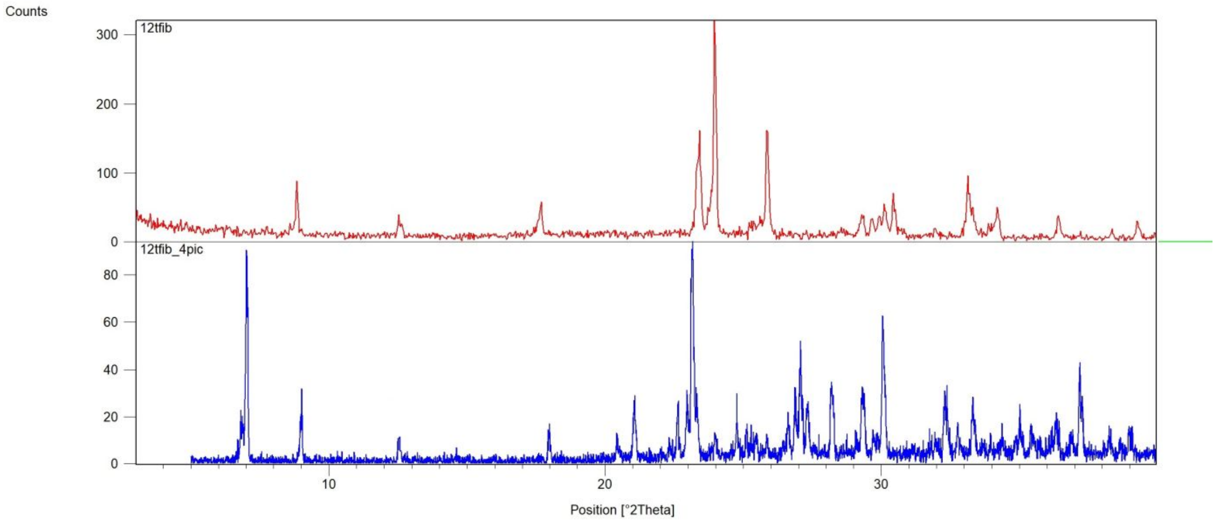

Figure S11. Comparison of the experimental XRPD pattern of the reaction mixture (**12tfib** + **4pic**) after grinding and XRPD pattern of the (**12tfib**).

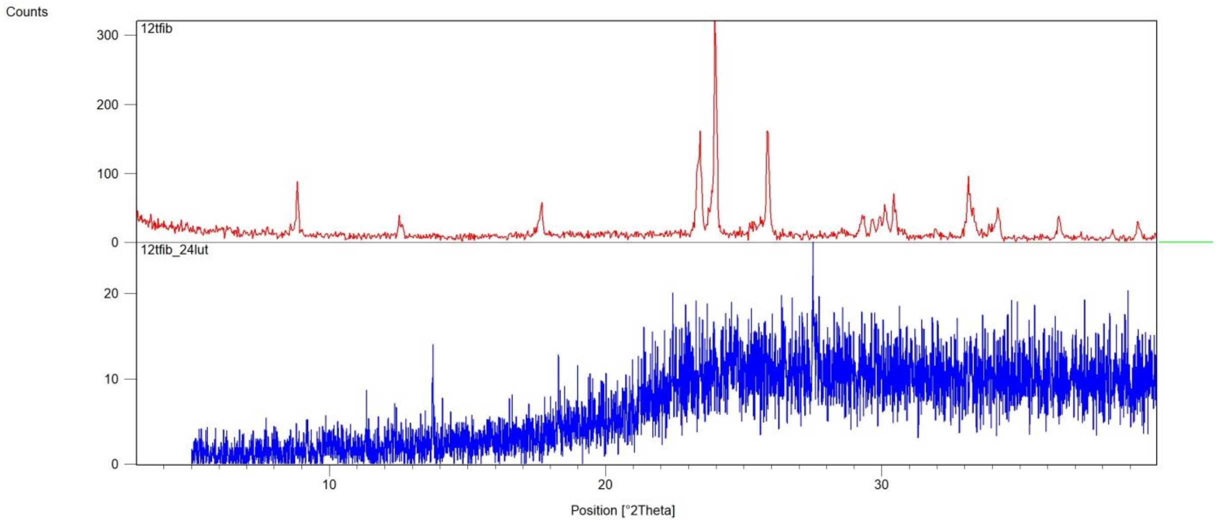

Figure S12. Comparison of the experimental XRPD pattern of the reaction mixture (**12tfib** + **24lut**) after grinding and XRPD pattern of the (**12tfib**).

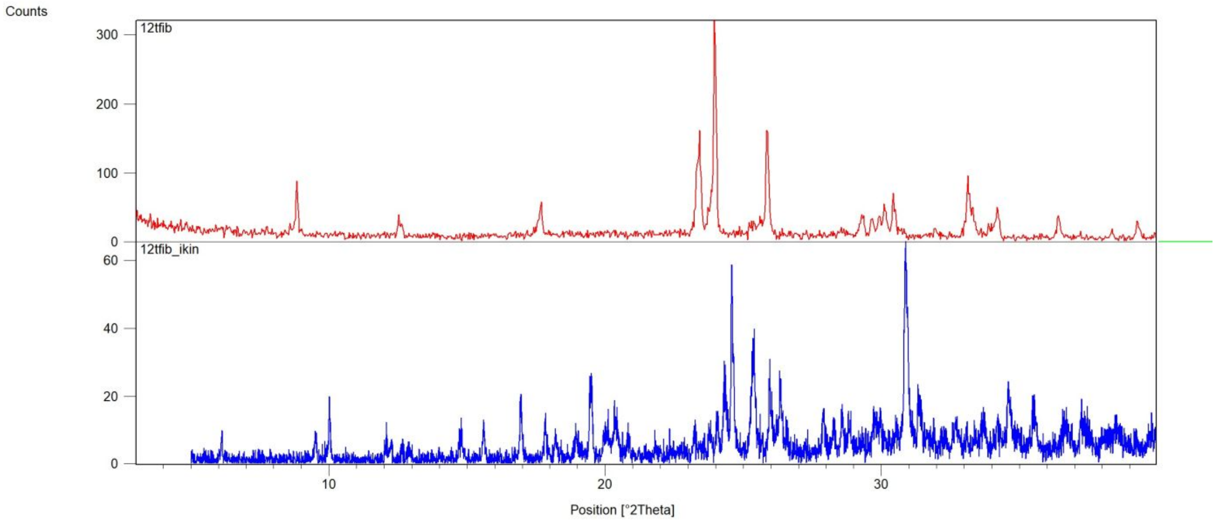

Figure S13. Comparison of the experimental XRPD pattern of the reaction mixture (**12tfib** + **iquin**) after grinding and XRPD pattern of the (**12tfib**).

Table S1. Volumes and masses of reactants used in grinding experiments.

acceptor	*m*(acceptor) / mg	*V*(acceptor) /μL	*m*(**12tfib**) / (mg)	D:A
2pic	-	33.30	68.50	1:2
**3pic**	**-**	**33.30**	**68.50**	**1:2**
4pic	-	33.20	68.50	1:2
24lut	-	37.90	67.24	1:2
26lut	-	38.58	67.24	1:2
34lut	-	37.20	67.24	1:2
35lut	-	37.00	65.20	1:2
246kol	-	40.05	62.90	1:2
3acpy	-	34.83	64.32	1:2
4acpy	-	35.41	64.32	1:2
kin	-	36.00	60.60	1:2
ikin	-	35.50	60.60	1:2
4cnpy	34.80	-	67.91	1:2
4bzpy	48.70	-	53.92	1:2
dmap	37.80	-	62.20	1:2

Table S2. Halogen bond lengths and angles in the prepared cocrystals.

compound	halogen bond	*d*(I···N) / Å	∢(C–I ···N) / °
**(12tfib)(3pic)**	I1···N1	2.775(2)	176.63(6)
	I2···N2	3.147(2)	167.72(8)
	I4···N2	3.059(1)	174.55(8)
**(12tfib)(26lut)**	I1···N1	2.913(4)	174.34(8)
**(12tfib)(34lut)**$_2$	I1···N1	2.875(4)	171.38(7)
	I2···N2	2.821(1)	178.53(6)
**(12tfib)(35lut)**	I1···N1	2.819(4)	177.26(7)
**(12tfib)(246col)**$_2$	I1···N1	2.943(3)	177.51(8)
	I2···N2	2.950(7)	172.44(7)
**(12tfib)(quin)**	I1···N1	2.977(4)	166.56(7)
	I2···N1	3.136(5)	165.81(4)
**(12tfib)(dmap)**$_2$	I1···N3	2.736(3)	174.46(9)
	I2···N1	2.984(4)	164.01(6)
	I3···N5	2.822(1)	175.60(7)
	I4···N7	2.852(4)	164.18(7)
**(12tfib)**$_2$**(dmap)**$_3$**(dmap)**	I1···N7	2.807(6)	178.19(7)
	I2···N014	2.786(6)	179.67(7)
	I3···N019	3.025(6)	173.82(5)
	I4···N7	2.965(7)	176.50(5)

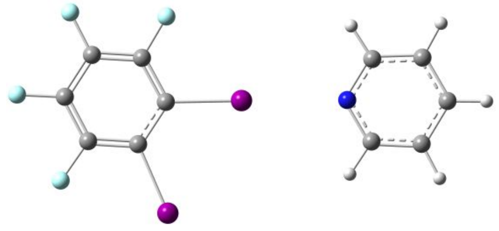

Figure S14. Optimized structure of the (**12tfib**)(**py**). $E = -1471.543779$ a.u.

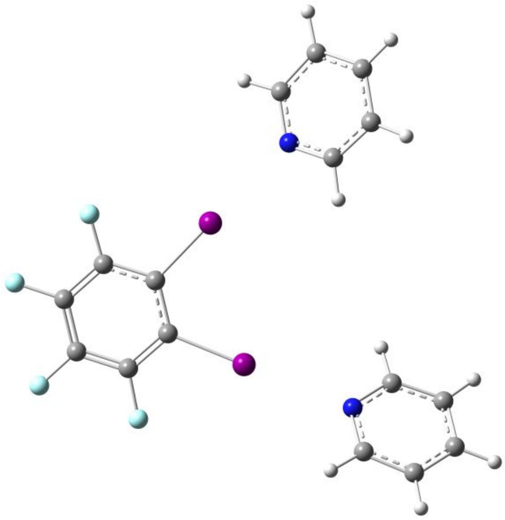

Figure S15. Optimized structure of the (**12tfib**)(**py**)$_2$. $E = -1719.818006$ a.u.

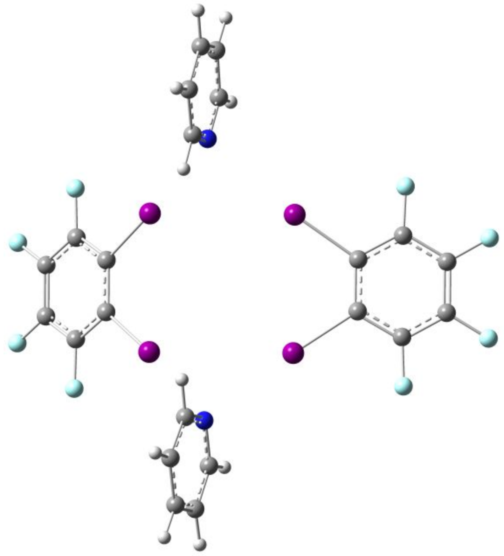

Figure S16. Optimized structure of the (**12tfib**)$_2$(**py**)$_2$. $E = -2943.096536$ a.u.

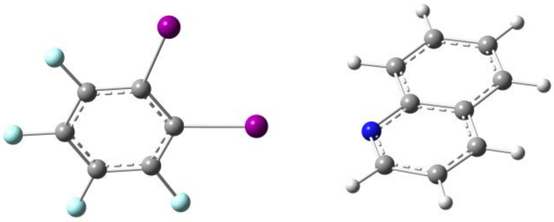

Figure S17. Optimized structure of the (**12tfib**)(**quin**). $E = -1625.180739$

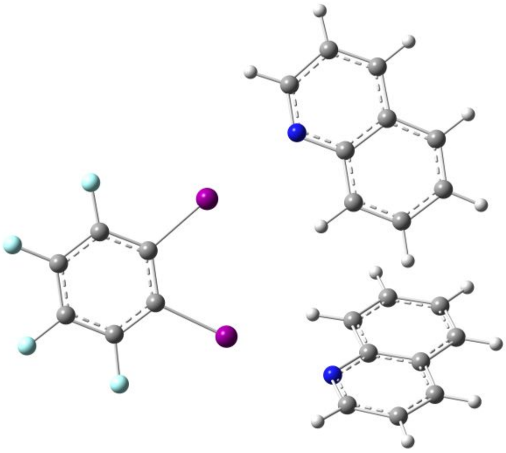

Figure S18. Optimized structure of the (**12tfib**)(**quin**)$_2$. $E = -2027.095869$ a.u.

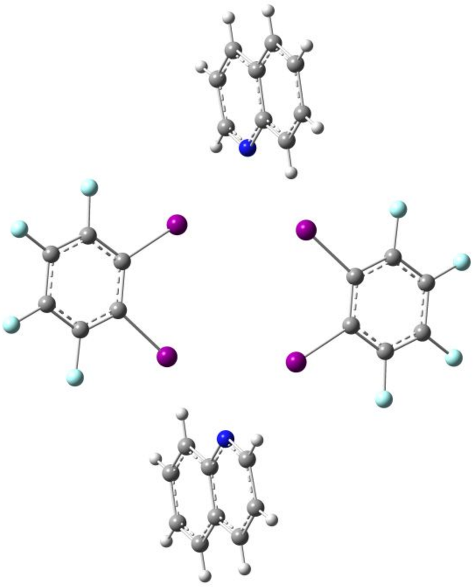

Figure S19. Optimized structure of the (**12tfib**)$_2$(**quin**)$_2$. $E = -3250.372476$ a.u.

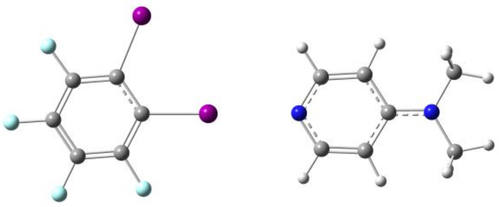

Figure S20. Optimized structure of the (**12tfib**)(**dmap**). $E = -1732.558697$ a.u.

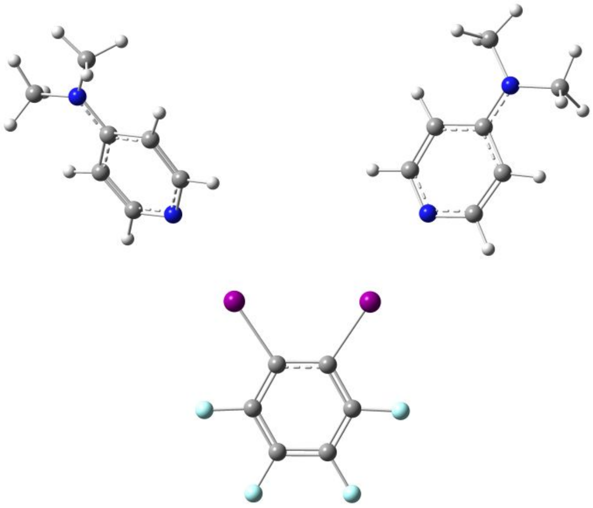

Figure S21. Optimized structure of the (**12tfib**)(**dmap**)$_2$. $E = -1987.744215$ a.u.

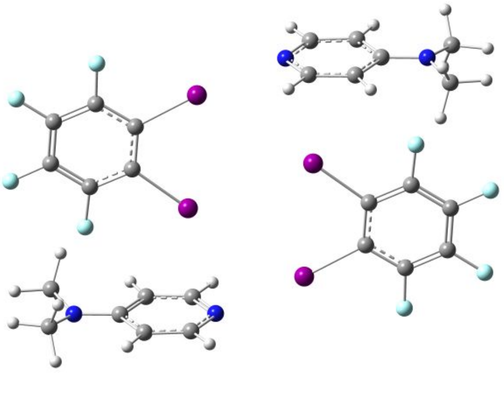

Figure S22. Optimized structure of the (**12tfib**)$_2$(**dmap**)$_2$. $E = -3975.007635$ a.u.

Table S3 Parameters of halogen bonds with nitrogen atom as a bifurcated acceptor.

	φ_1 / °	φ_2 / °	ψ / °	χ_1 / °	χ_2 / °	δ / °	***d*(I1···N)/Å**	***d*(I2···N)/Å**	***d*(I1···I2)/Å**
ANUPOP	175.694	174.57	84.096	50.022	57.799	74.125	3.117	3.162	4.024
CEQLIW	171.209	158.346	89.192	51.552	26.128	114.89	2.896	3.306	4.877
	171.701	158.726	89.753	46.994	27.944	114.482	2.908	3.269	4.793
	172.86	151.012	89.713	52.157	31.356	113.823	2.884	3.392	5.033
	155.016	173.21	86.92	31.774	51.73	112.561	3.38	2.87	4.948
EFUFES	174.397	165.17	84.772	71.691	5.102	111.928	2.848	3.255	4.5
ENAKAH	154.828	152.987	10.323	89.87	87.779	78.374	3.409	3.472	4.518
LAGGOT	147.57	159.366	85.119	35.569	31.218	113.31	3.501	3.343	4
LAGHAG	165.988	152.405	85.53	73.17	26.339	125.426	3.132	3.322	4.97
	164.714	146.08	85.293	74.269	36.763	120.041	3.153	3.428	5.203
LEZXEW	131.704	155.757	60.701	32.076	39.536	114.286	3.525	3.292	4.399
OJIKEZ	177.63	141.008	74.965	60.885	7.841	111.363	2.793	3.484	4.007
PERLEG	172.285	176.242	80.3	11.191	86.545	83.443	3.396	2.871	4.293
QEFNAU	163.935	161.407	82.539	56.263	24.344	113.249	3.214	3.185	4.331
	142.623	124.296	74.279	57.271	23.071	113.249	3.215	3.47	4.331
VIHFIC	173.624	168.218	81.658	53.217	33.988	100.153	2.901	3.123	4.242
YANPIP	170.375	136.007	82.107	50.314	41.663	95.115	2.928	3.391	4.042
	140.725	178.477	76.368	39.669	48.299	95.425	3.488	2.879	4.019
	142.215	169.242	81.088	39.35	36.769	106.289	3.463	3.002	4.066
	138.874	171.88	79.338	41.55	48.752	95.115	3.431	3.086	4.042
BONBIR02	149.583	149.583	90	87.286	87.286	174.572	3.516	3.516	5.89
CEZVEL	152.606	174.913	89.044	61.8	41.263	79.611	3.49	3.135	4.551
DIXXEO	175.994	169.309	89.029	87.517	8.72	100.666	2.931	3.408	4.676
DIXXOY	174.069	180	82.391	13.581	90	88.579	3.484	3.06	4.605
	174.069	174.069	82.391	90	90	177.159	3.484	3.484	6.968
EFUGIX	171.152	160.702	74.755	87.739	31.915	60.71	3.029	3.494	4.026
	164.038	170.348	69.738	19.325	79.94	60.816	3.441	3.042	3.967
ISODOK	164.062	147.982	79.358	88.152	20.446	95.054	3.046	3.5	4.638
LAGHEK	173.506	155.659	77.744	21.048	68.701	90.3	3.075	3.521	4.23
NAHSIB	149.99	177.452	84.552	32.516	89.559	120.05	3.393	2.603	4.536
QICQIG	165.377	172.854	74.986	22.883	58.205	105.13	3.239	2.877	4.161
SODBET	176.68	178.706	75.061	89.717	14.097	86.993	2.923	3.443	4.245
TAWFEF	144.554	164.745	82.788	39.757	42.09	99.682	3.406	3.13	4.06
	144.114	165.693	78.646	37.322	42.735	101.256	3.471	3.024	4.023
TEPKUW	167.824	164.2	89.705	80.715	15.272	84.028	3.312	3.239	4.116
TIPKEL	175.915	162.703	82.852	66.172	3.923	110.542	2.869	3.206	4.482
TOJCEB	165.85	165.85	64.636	40.182	40.182	112.214	3.121	3.121	4.182
TUBMUB	146.681	175.891	78.503	30.989	87.416	117.698	3.437	3.109	4.442
UDUJOW	166.337	159.94	82.731	56.42	71.447	54.007	3.096	3.141	3.908

VEBMU O	171.548	170.699	78.659	84.824	15.785	80.829	2.945	3.391	4.386
VIXXAE	135.761	170.398	84.34	62.058	67.729	62.032	3.521	3.342	3.958
YAFKOI	177.785	174.267	84.11	6.706	85.484	90.029	3.519	3.003	4.654
YIQYOP	168.528	158.318	81.203	35.585	51.104	94.895	3.148	3.402	4.121
ZIXYAK	172.054	173.353	84.089	79.826	6.814	102.734	2.909	3.241	4.323

LUKWAS02	164.2	101.018	65.182	86.849	84.623	141.435	3.04	3.441	5.847
LUKWOG	175.704	67.636	59.588	88.534	87.413	143.278	2.839	3.471	3.909
LUKWOG01	164.485	103.896	85.934	85.802	89.535	140.074	2.954	3.444	5.828

E(4cnpy) = −340.51178379 a.u.

N	2.1838990	0.0001440	0.0001350
C	1.4973200	1.1384260	-0.0000430
H	2.0809730	2.0517820	-0.0007840
C	0.1119690	1.1987440	-0.0000600
H	-0.4068710	2.1467220	-0.0006190
C	-0.5914090	-0.0002180	0.0010260
C	0.1121780	-1.1988740	0.0000780
H	-0.4062660	-2.1470620	-0.0005280
C	1.4976760	-1.1381300	-0.0003680
H	2.0813980	-2.0514720	-0.0002830
C	-2.0288750	-0.0003410	-0.0001510
N	-3.1756690	0.0001970	-0.0002330

E(12tfib) = −1223.26795206 a.u.

Symbol	X	Y	Z
C	0.2957480	-0.6982830	-0.0000490
C	0.2957600	0.6982870	-0.0000440
C	1.5066980	1.3737640	0.0000210
C	2.7111140	0.6907680	0.0000070
C	2.7111040	-0.6907700	-0.0000430
C	1.5066900	-1.3737600	-0.0000470
F	3.8561390	-1.3534230	-0.0000270
F	3.8561490	1.3534100	0.0000750
F	1.5540980	-2.6997640	-0.0000710
F	1.5541040	2.6997670	0.0000720
I	-1.4296900	-1.8544040	0.0000330
I	-1.4296900	1.8544050	-0.0000230

E(12tfib·4cnpy) = −1563.78794476 a.u.

Symbol	X	Y	Z
N	-3.5262510	0.3042670	0.0003560
C	-4.2997190	1.3854490	0.0001710
H	-3.7894490	2.3418130	0.0002370
C	-5.6842350	1.3262890	-0.0000880
H	-6.2816660	2.2266340	-0.0002220
C	-6.2826730	0.0712400	-0.0001660
C	-5.4820080	-1.0655960	0.0000190
H	-5.9198050	-2.0534720	-0.0000370
C	-4.1073650	-0.8913560	0.0002790
H	-3.4436650	-1.7485010	0.0004380
C	1.5281460	0.6948620	0.0000390
I	-0.5555030	0.5405940	0.0002180
C	2.3961690	-0.3997660	-0.0000270
I	1.7539810	-2.3773910	-0.0000130
C	3.7657710	-0.1884180	-0.0001340
F	4.6218840	-1.2032800	-0.0001950
C	4.2932180	1.0920900	-0.0001840
F	5.6042100	1.2765550	-0.0002890
C	3.4403860	2.1780070	-0.0001260
F	3.9324860	3.4070790	-0.0001750
C	2.0711750	1.9698780	-0.0000110
F	1.2926490	3.0473660	0.0000510
C	-7.7152150	-0.0501160	-0.0004250
N	-8.8577110	-0.1472800	-0.0006290

E(12tfib·4cnpy$_2$) = −1904.30709210 a.u.

Symbol	X	Y	Z
N	-3.6238200	-1.7295350	-0.0237290
C	-4.5869690	-1.8449130	0.8852280
H	-4.6420960	-1.0586640	1.6295050
C	-5.4870510	-2.8985350	0.9066410
H	-6.2577360	-2.9595590	1.6615580
C	-5.3688910	-3.8761630	-0.0748760
C	-4.3660120	-3.7635490	-1.0313850
H	-4.2519810	-4.5074600	-1.8067690
C	-3.5199320	-2.6684320	-0.9590850
H	-2.7246140	-2.5386920	-1.6841500
N	3.5752890	-1.8252270	0.0624540
C	4.6674850	-1.8358960	-0.6952810
H	4.8458970	-0.9495420	-1.2934760
C	5.5490630	-2.9042950	-0.7397100
H	6.4276410	-2.8777430	-1.3681810
C	5.2687950	-4.0132930	0.0506070
C	4.1296790	-4.0113150	0.8479940
H	3.8880870	-4.8588250	1.4732680
C	3.3165560	-2.8895480	0.8156560
H	2.4187520	-2.8432050	1.4212830
C	-0.6671570	2.4229690	-0.0053040
I	-1.8588780	0.7056570	-0.0082200
C	0.7301770	2.4031440	0.0074530
I	1.8720220	0.6527740	0.0370520
C	1.4190830	3.6053100	0.0033160
F	2.7486970	3.6381660	0.0141410
C	0.7559500	4.8208910	-0.0121940
F	1.4362720	5.9578370	-0.0158200
C	-0.6244380	4.8404210	-0.0233680
F	-1.2720840	5.9962100	-0.0377510

C	-1.3217920	3.6440900	-0.0200590
F	-2.6498200	3.7144920	-0.0318340
C	6.1475330	-5.1510900	0.0435820
C	-6.2742490	-4.9926310	-0.1006460
N	-6.9953130	-5.8839490	-0.1207410
N	6.8473980	-6.0593530	0.0375600

E(dmap) = −382.22741093 a.u.

Symbol	X	Y	Z
N	2.6524750	0.0000010	0.0190080
C	1.9438550	1.1265400	0.0084150
H	2.5153540	2.0491460	0.0212510
C	0.5631770	1.1923320	-0.0146130
H	0.0827630	2.1590100	-0.0167530
C	-0.1809520	0.0000000	-0.0330970
N	-1.5465380	-0.0000010	-0.0675600
C	-2.2635080	1.2519020	0.0266970
H	-2.0055020	1.9168820	-0.8011670
H	-2.0505660	1.7762310	0.9645280
H	-3.3310470	1.0556620	-0.0216170
C	-2.2635240	-1.2518960	0.0266580
H	-2.0054630	-1.9168830	-0.8011810
H	-3.3310590	-1.0556480	-0.0217450
H	-2.0506640	-1.7762230	0.9645100
C	0.5631820	-1.1923370	-0.0145940
H	0.0827650	-2.1590130	-0.0167150
C	1.9438530	-1.1265460	0.0084460
H	2.5153600	-2.0491450	0.0212780

E(12tfib·dmap) = −1605.50749715 a.u.

Symbol	X	Y	Z
N	-3.0408490	0.3129520	0.0025880
C	-3.8312980	1.3849240	0.0031130
H	-3.3291950	2.3468110	0.0044890
C	-5.2099100	1.3350200	0.0019200
H	-5.7686860	2.2582360	0.0022880
C	-5.8539450	0.0831320	0.0001300
N	-7.2098280	-0.0277620	-0.0009630
C	-8.0299800	1.1642930	-0.0019560
H	-7.8440390	1.7775540	-0.8883130
H	-7.8448860	1.7781210	0.8841400
H	-9.0771840	0.8761690	-0.0022540
C	-7.8252920	-1.3372420	-0.0043590
H	-7.5424520	-1.9103080	-0.8920080
H	-8.9053980	-1.2231100	-0.0040230
H	-7.5425300	-1.9144970	0.8805430
C	-5.0152000	-1.0476690	-0.0005960
H	-5.4168710	-2.0492970	-0.0022210
C	-3.6468540	-0.8730080	0.0007050
H	-2.9951400	-1.7406700	0.0001570
C	1.9049940	0.6954480	0.0000010
I	-0.1873790	0.5417500	0.0013550
C	2.7754320	-0.3972420	-0.0001860
I	2.1305160	-2.3755990	0.0002540
C	4.1456030	-0.1898770	-0.0008590
F	5.0013490	-1.2065220	-0.0010360
C	4.6747070	1.0895080	-0.0013950
F	5.9872740	1.2731610	-0.0020100
C	3.8223980	2.1757240	-0.0012770
F	4.3171090	3.4051350	-0.0017770
C	2.4532010	1.9683020	-0.0005890

F	1.6777950	3.0494190	-0.0004400

E(12tfib·dmap$_2$) = −1987.74421466 a.u.

Symbol	X	Y	Z
N	-3.4857230	-1.4495350	0.0331380
C	-4.7554470	-1.5307120	0.4254620
H	-5.1983810	-0.6125310	0.7971610
C	-5.5118410	-2.6844080	0.3837620
H	-6.5355560	-2.6540640	0.7241160
C	-4.9311960	-3.8707210	-0.1018360
N	-5.6290510	-5.0384780	-0.1681050
C	-7.0065780	-5.0797000	0.2712360
H	-7.6336100	-4.3983110	-0.3110060
H	-7.0984970	-4.8103800	1.3274190
H	-7.3913620	-6.0874240	0.1433910
C	-4.9895270	-6.2308850	-0.6787990
H	-4.6540480	-6.0947150	-1.7109820
H	-5.6995990	-7.0526550	-0.6586730
H	-4.1236130	-6.5125600	-0.0725010
C	-3.5890630	-3.7821980	-0.5156120
H	-3.0591680	-4.6390240	-0.9027350
C	-2.9337650	-2.5706230	-0.4262850
H	-1.8990060	-2.4951800	-0.7449620
N	3.5211460	-1.1634700	-0.0117150
C	4.6389760	-1.1566180	-0.7351230
H	4.7991570	-0.2802840	-1.3548050
C	5.5738350	-2.1718610	-0.7351080
H	6.4525330	-2.0770250	-1.3544940
C	5.3607890	-3.3033630	0.0738890
N	6.2486300	-4.3353380	0.1154110
C	7.4589210	-4.2753250	-0.6745460
H	8.0808980	-3.4204110	-0.3930770

H	7.2352780	-4.1982590	-1.7422510
H	8.0347870	-5.1822170	-0.5138790
C	5.9969340	-5.4665250	0.9807160
H	5.9348450	-5.1638780	2.0302550
H	6.8098710	-6.1799420	0.8803660
H	5.0644160	-5.9727870	0.7154830
C	4.1792250	-3.3077550	0.8382810
H	3.9312860	-4.1308480	1.4908740
C	3.3175590	-2.2326020	0.7551180
H	2.4060570	-2.2292180	1.3439940
C	-0.8605370	2.8296390	0.0194470
I	-1.9697510	1.0512780	0.0428670
C	0.5367260	2.8765730	-0.0066840
I	1.7648500	1.1772630	-0.0141550
C	1.1658510	4.1109770	-0.0258200
F	2.4937770	4.2101160	-0.0518200
C	0.4466640	5.2938640	-0.0187310
F	1.0720290	6.4645280	-0.0372760
C	-0.9325780	5.2476510	0.0084460
F	-1.6351500	6.3737570	0.0161200
C	-1.5708210	4.0192550	0.0270140
F	-2.9024700	4.0292400	0.0523620

$E(\cdot py) = -248.26566016$ a.u.

Symbol	X	Y	Z
C	1.1913520	0.6688560	-0.0000030
C	-0.0002350	1.3764160	-0.0000460
C	-1.1915550	0.6685240	0.0000280
C	-1.1362720	-0.7187020	-0.0000290
C	1.1365270	-0.7183180	0.0000630
H	2.1465060	1.1761080	0.0000060
H	-0.0003530	2.4589650	-0.0000030
H	-2.1469050	1.1754080	0.0001160
H	-2.0500020	-1.3034360	0.0001130
H	2.0503950	-1.3028260	0.0000310
N	0.0002080	-1.4092680	-0.0000490

E(12tfib·py) = −1471.54377867 a.u.

Symbol	X	Y	Z
N	-3.9413740	0.2830870	0.0003920
C	-4.7372010	1.3485760	-0.0000740
H	-4.2433090	2.3141300	0.0000850
C	-6.1213540	1.2579230	-0.0007450
H	-6.7248260	2.1550150	-0.0010950
C	-5.8766990	-1.1159600	-0.0004800
H	-6.2845510	-2.1172760	-0.0006090
C	-4.5033360	-0.9222610	0.0001970
H	-3.8226380	-1.7667620	0.0005930
C	1.0581800	0.6984420	0.0001170
I	-1.0290730	0.5404150	0.0004620
C	1.9293580	-0.3937200	-0.0000620
I	1.2890830	-2.3727370	0.0000220
C	3.2988550	-0.1819470	-0.0002920
F	4.1565970	-1.1962040	-0.0004670
C	3.8248790	1.0989210	-0.0003450
F	5.1363740	1.2852810	-0.0005650
C	2.9704170	2.1834760	-0.0001720
F	3.4616930	3.4135920	-0.0002170
C	1.6015580	1.9733530	0.0000530
F	0.8227860	3.0512670	0.0002100
C	-6.7005600	-0.0012690	-0.0009520
H	-7.7772540	-0.1123190	-0.0014890

E(12tfib·py$_2$) = −1719.81800583 a.u.

Symbol	X	Y	Z
N	3.2219960	-2.6683880	-0.0212540
C	3.7170160	-3.5858720	0.8024440
H	3.0242620	-4.0015830	1.5254270
C	5.0391460	-4.0060960	0.7615430
H	5.3916720	-4.7562190	1.4559220
C	5.3728190	-2.4818860	-1.0440550
H	5.9925190	-2.0142580	-1.7963590
C	4.0367770	-2.1302670	-0.9240280
H	3.5963660	-1.3861020	-1.5788400
N	1.1399260	4.0208920	0.0391980
C	0.7747190	5.2332240	-0.3673360
H	-0.2394910	5.3271790	-0.7391760
C	1.6219830	6.3311940	-0.3274670
H	1.2781460	7.2972330	-0.6702020
C	3.2934500	4.8947820	0.5876540
H	4.2857810	4.7114520	0.9760130
C	2.3734310	3.8594700	0.5072910
H	2.6334770	2.8580000	0.8331070
C	-1.6365910	-1.2376790	0.0032130
I	0.3699990	-1.8344350	0.0148650
C	-2.0534310	0.0962040	-0.0069030
I	-0.7417640	1.7282610	0.0023980
C	-3.4112420	0.3744920	-0.0178590
F	-3.8590720	1.6278010	-0.0246540
C	-4.3593570	-0.6350610	-0.0202550
F	-5.6533380	-0.3444440	-0.0305120
C	-3.9469020	-1.9521060	-0.0109500
F	-4.8441400	-2.9287590	-0.0129480
C	-2.5927980	-2.2402450	0.0001660
F	-2.2462780	-3.5251370	0.0083890

C	2.9075030	6.1559490	0.1601350
H	3.5970690	6.9889990	0.2064750
C	5.8826960	-3.4407710	-0.1819120
H	6.9204190	-3.7421140	-0.2454840

E(quin) = −401.90239690 a.u.

Symbol	X	Y	Z
C	-2.3980870	0.6999040	0.0000230
C	-1.2254960	1.3998230	0.0001910
C	0.0147840	0.7183630	0.0001310
C	0.0281900	-0.6981600	-0.0001170
C	-1.2022440	-1.3976800	-0.0002970
C	-2.3845290	-0.7137680	-0.0002240
H	1.2839940	2.4691460	0.0005080
H	-3.3445230	1.2244880	0.0000810
H	-1.2259860	2.4834550	0.0003840
C	1.2608770	1.3855210	0.0003060
H	-1.1656470	-2.4789780	-0.0004890
H	-3.3223760	-1.2540030	-0.0003600
C	2.3116700	-0.7604030	-0.0000680
C	2.4094220	0.6513870	0.0002170
H	3.2185500	-1.3579210	-0.0000820
H	3.3829720	1.1219920	0.0003600
N	1.1836430	-1.4168710	-0.0001960

E(12tfib·quin) = −1625.18073915 a.u.

Symbol	X	Y	Z
C	6.2787030	-0.4336320	2.1008260
H	7.0397390	-0.6571970	2.8367260
C	4.2779760	0.1401940	0.2327130
N	3.2807600	0.4163190	-0.6512410
C	4.9225770	-0.3791410	2.4955690
H	4.6627090	-0.5616840	3.5300810
C	5.6361280	0.0868060	-0.1651670
C	6.6279170	-0.2059790	0.8004620
H	7.6648550	-0.2454240	0.4890720
C	4.9183780	0.6081580	-2.3934230
H	5.1042350	0.8020320	-3.4405510
C	3.9430460	-0.0994070	1.5858840
H	2.8990900	-0.0530250	1.8680960
C	3.5947920	0.6378720	-1.8993420
H	2.7708970	0.8545730	-2.5730710
C	5.9326560	0.3325850	-1.5248230
H	6.9635760	0.2985190	-1.8574100
C	-3.9231800	-0.2267810	0.1469380
I	0.3783060	0.5696620	-0.1790510
I	-1.8931570	-2.3760670	-0.1806910
F	-1.4944950	3.0421680	0.1537510
F	-4.7659840	-1.2533680	0.1515000
F	-5.7703090	1.2045120	0.4070760
F	-4.1275100	3.3573470	0.4076490
C	-1.7022840	0.6911850	0.0131150
C	-2.5567190	-0.4143880	0.0134590
C	-2.2575590	1.9536390	0.1477410
C	-4.4618640	1.0418240	0.2806100
C	-3.6236730	2.1389810	0.2808130

E(12tfib·quin$_2$) = −2027.09586867 a.u.

Symbol	X	Y	Z
C	4.9880930	-1.6471150	-1.7425190
H	5.8474770	-1.2219400	-2.2441810
C	2.7373820	-2.7106330	-0.4654290
N	1.6215580	-3.1996930	0.1428900
C	3.6887150	-1.2586880	-2.1380120
H	3.5691450	-0.5360080	-2.9344210
C	4.0396960	-3.1066110	-0.0710510
C	5.1607230	-2.5515200	-0.7328390
H	6.1535120	-2.8582450	-0.4253400
C	3.0209640	-4.5265850	1.5712320
H	3.0639750	-5.2451550	2.3777700
C	2.5874370	-1.7752730	-1.5164270
H	1.5846120	-1.4824550	-1.8000620
C	1.7666650	-4.0625300	1.1119440
H	0.8545530	-4.4254930	1.5760020
C	4.1511190	-4.0454280	0.9802080
H	5.1346570	-4.3686760	1.3009320
C	5.0814950	1.3448140	0.6671040
H	6.0134340	0.8105660	0.7997460
C	2.6452930	2.6773570	0.3206200
N	1.4450780	3.2983920	0.1520170
C	3.8620690	0.6319200	0.7006610
H	3.8777020	-0.4401170	0.8536860
C	3.8672430	3.3945830	0.2900650
C	5.0848940	2.6958750	0.4665720
H	6.0144030	3.2521100	0.4399980
C	2.5928320	5.3921790	-0.0827820
H	2.5039830	6.4574320	-0.2442660
C	2.6706090	1.2776970	0.5299770
H	1.7303160	0.7398610	0.5455600

C	1.4300100	4.5900780	-0.0391130
H	0.4539090	5.0477150	-0.1695450
C	3.8055010	4.7907840	0.0810090
H	4.7250310	5.3639680	0.0542100
C	-4.0936220	1.2883260	-0.0208660
I	-1.0568360	-1.8567770	-0.0184830
I	-1.1421740	1.8287480	0.0606230
F	-4.0445950	-2.7832110	-0.0872710
F	-4.1768600	2.6162250	-0.0003700
F	-6.4459620	1.2141190	-0.0726850
F	-6.3795460	-1.4927260	-0.1149280
C	-2.8332560	-0.7512540	-0.0280070
C	-2.8669040	0.6463520	-0.0049720
C	-4.0265340	-1.4530100	-0.0646990
C	-5.2823280	0.5787040	-0.0581550
C	-5.2485950	-0.8014530	-0.0796160

E(12tfib$_2$·py$_2$) = −2943.09653557 a.u.

Symbol	X	Y	Z
C	-4.6897400	1.4476860	-1.2110450
I	-2.0718140	1.9356400	0.1820300
F	-4.7425040	-2.6358860	-1.2613680
F	-4.7338010	2.7820220	-1.2276670
F	-6.7741350	1.4435640	-2.3196990
F	-6.7786830	-1.2772870	-2.3365950
C	-3.6180580	-0.6342530	-0.6521270
C	-3.6149830	0.7704780	-0.6429100
C	-4.6944490	-1.3020670	-1.2283100
C	-5.7591830	0.7704320	-1.7835970
C	-5.7615530	-0.6140630	-1.7922420
N	0.0219540	3.8155520	1.4314230
C	0.2557910	6.2067080	1.4540330
H	0.3216960	7.1283880	0.8866680
C	0.1081830	4.9891790	0.7934640
H	0.0570320	4.9466500	-0.2921370
C	0.3162520	6.2038460	2.8454840
H	0.4313610	7.1326110	3.3954400
C	4.6905130	-1.4477450	-1.2102560
I	2.0802820	1.8143770	0.1574710
F	4.7429820	2.6358180	-1.2610030
F	4.7346690	-2.7820850	-1.2267590
F	6.7748820	-1.4435880	-2.3189670
F	6.7792290	1.2772640	-2.3361350
C	3.6186850	0.6341660	-0.6515150
C	3.6157180	-0.7705640	-0.6421560
C	4.6950180	1.3019940	-1.2277970
C	5.7598940	-0.7704760	-1.7829010
C	5.7621610	0.6140180	-1.7916850
C	0.0802620	3.8229920	2.7684700

H	0.0069380	2.8527240	3.2541740
C	0.2264150	4.9876670	3.5186340
H	0.2685860	4.9358860	4.6009440
I	2.0726360	-1.9358220	0.1828750
I	-2.0796930	-1.8144100	0.1569500
C	-0.1091820	-4.9889220	0.7934930
C	-0.0811440	-3.8228650	2.7685780
C	-0.2311180	-4.9872080	3.5185000
C	-0.3229680	-6.2031450	2.8451840
C	-0.2606140	-6.2061110	1.4538170
H	-0.0564350	-4.9464790	-0.2920340
H	-0.0061390	-2.8527920	3.2544140
H	-0.2746430	-4.9353640	4.6007540
H	-0.4410550	-7.1316460	3.3949520
H	-0.3279620	-7.1276160	0.8863350
N	-0.0209990	-3.8155300	1.4316120

E(12tfib$_2$·dmap$_2$) = −2970.97620860 a.u.

Symbol	X	Y	Z
C	3.9503990	-3.5544690	-0.0008350
I	0.9450370	-3.2492390	0.0008860
F	5.0969080	0.3678390	-0.0014330
F	3.6465740	-4.8544350	-0.0006950
F	6.2312800	-4.1727760	-0.0020740
F	6.9592940	-1.5467060	-0.0024420
C	3.3344760	-1.2332240	-0.0004260
C	2.9515090	-2.5848140	-0.0002580
C	4.6868810	-0.9089010	-0.0011940
C	5.3000780	-3.2220760	-0.0015610
C	5.6706310	-1.8878300	-0.0017420
N	-1.8868850	-3.9268640	0.0015840
C	-3.9458600	-3.5502860	-1.1999990
H	-4.4133820	-3.4472740	-2.1711790
C	-2.5855200	-3.8042010	-1.1359680
H	-2.0142730	-3.8972430	-2.0574050
C	-4.6802030	-3.4011150	-0.0014280
C	-3.9504140	3.5545300	-0.0010160
I	-1.9744340	-0.3606880	0.0027810
F	-5.0971100	-0.3677470	0.0006590
F	-3.6465150	4.8544730	-0.0016480
F	-6.2312690	4.1729320	-0.0029160
F	-6.9594190	1.5469330	-0.0017940
C	-3.3346260	1.2332480	0.0008600
C	-2.9515780	2.5848150	0.0002460
C	-4.6870450	0.9089800	0.0001560
C	-5.3001040	3.2221940	-0.0017040
C	-5.6707350	1.8879640	-0.0011260
C	-2.5882210	-3.8055570	1.1375970
H	-2.0191600	-3.8996900	2.0602690

C	-3.9487040	-3.5516200	1.1986940
H	-4.4185340	-3.4497970	2.1688830
I	-0.9450770	3.2491060	0.0007210
I	1.9742200	0.3606880	0.0003690
C	2.5868800	3.8051870	-1.1363250
C	2.5874280	3.8043710	1.1372560
C	3.9479300	3.5506850	1.1994040
C	4.6805300	3.4013140	-0.0001620
C	3.9473100	3.5515390	-1.1993030
H	2.0164620	3.8990600	-2.0581830
H	2.0175040	3.8975680	2.0594960
H	4.4168470	3.4481110	2.1699540
H	4.4158610	3.4497350	-2.1701100
N	1.8871450	3.9264880	0.0007080
N	6.0188590	3.1221880	-0.0006390
C	6.6700860	2.7817470	1.2544570
H	7.7167700	2.5519560	1.0603700
H	6.6365580	3.6253080	1.9507970
H	6.2052860	1.9090530	1.7300210
C	6.6693970	2.7825250	-1.2562910
H	7.7160590	2.5520910	-1.0628610
H	6.2040330	1.9104090	-1.7323670
H	6.6359040	3.6266700	-1.9519390
N	-6.0185060	-3.1219590	-0.0029020
C	-6.6679830	-2.7814660	-1.2588750
H	-7.7148590	-2.5513790	-1.0661890
H	-6.2023660	-1.9089200	-1.7339090
H	-6.6337340	-3.6250850	-1.9551220
C	-6.6710440	-2.7829140	1.2518770
H	-7.7175720	-2.5530910	1.0569910
H	-6.6380340	-3.6271630	1.9474070
H	-6.2068860	-1.9106040	1.7287810

E(12tfib$_2$·quin$_2$) = −3250.37247569 a.u.

Symbol	X	Y	Z
C	-4.0326440	5.5120350	-2.3566760
H	-4.5304930	6.0278410	-3.1667250
C	-2.7231310	4.1519700	-0.2957540
N	-2.0704930	3.4685510	0.6848720
C	-3.1017020	4.4899100	-2.6499370
H	-2.8989400	4.2360500	-3.6818900
C	-3.6561740	5.1741710	0.0013230
C	-4.3041750	5.8471150	-1.0608550
H	-5.0170090	6.6283850	-0.8272150
C	-3.2316560	4.7735040	2.3286100
H	-3.3876900	4.9699710	3.3796960
C	-2.4596440	3.8230730	-1.6454590
H	-1.7435960	3.0371540	-1.8494940
C	-2.3190670	3.7713470	1.9320370
H	-1.7789850	3.2032880	2.6833950
C	-3.8955470	5.4701070	1.3621570
H	-4.6054670	6.2477310	1.6179350
C	4.9941950	1.4746440	-0.2485090
I	0.8522110	2.7932370	0.2809680
I	2.6921310	-0.4122380	-0.3166990
F	3.0448610	4.9876310	0.4093040
F	5.6914850	0.3639410	-0.4635650
F	7.0265130	2.6590650	-0.2559250
F	5.6990610	4.9789910	0.1817980
C	2.9265990	2.6456540	0.0902220
C	3.6128250	1.4495530	-0.1348000
C	3.6495610	3.8235700	0.1943140
C	5.7060100	2.6580190	-0.1436120
C	5.0296210	3.8400340	0.0793050
C	4.0382440	-5.5174210	2.3512900

H	4.5377170	-6.0347550	3.1593620
C	2.7245870	-4.1534850	0.2955720
N	2.0699880	-3.4682210	-0.6824610
C	3.1077760	-4.4959640	2.6483480
H	2.9069960	-4.2441280	3.6811840
C	3.6571490	-5.1750100	-0.0053090
C	4.3072820	-5.8499540	1.0542910
H	5.0197420	-6.6306890	0.8177390
C	3.2280830	-4.7698680	-2.3309870
H	3.3820920	-4.9642720	-3.3827550
C	2.4636990	-3.8272360	1.6464200
H	1.7479740	-3.0417890	1.8533830
C	2.3161750	-3.7685670	-1.9306930
H	1.7746060	-3.1990960	-2.6799080
C	3.8939140	-5.4682800	-1.3671800
H	4.6034180	-6.2453300	-1.6258430
C	-4.9953930	-1.4730610	0.2406810
I	-0.8519570	-2.7939270	-0.2714930
I	-2.6922540	0.4119360	0.3232650
F	-3.0456500	-4.9863020	-0.4145250
F	-5.6928380	-0.3622440	0.4546530
F	-7.0286090	-2.6559510	0.2365110
F	-5.7008810	-4.9760760	-0.1993500
C	-2.9271310	-2.6449860	-0.0907560
C	-3.6134970	-1.4487900	0.1333600
C	-3.6504830	-3.8221720	-0.2002870
C	-5.7076030	-2.6556970	0.1302630
C	-5.0310740	-3.8378100	-0.0917140

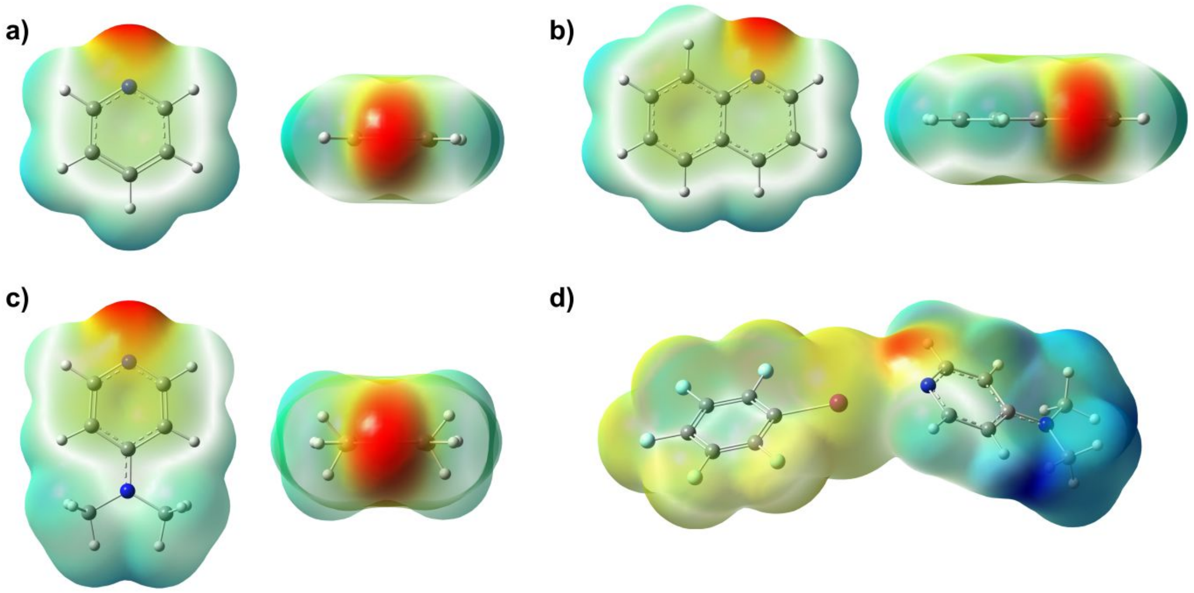

Figure S23. Molecular electrostatic potential plotted on isodensity surface (0.001 a.u.) for a) pyridine (side and front view); b) quinoline (side and front view); c) 4-*N*,*N*'-dimethylaminopyridine (side and front view); d) (**ipfb**)(**dmap**) molecular complex in which angle between mean planes of **ipfb** and **dmap** molecules is 36°.
